# Abstracts from The 8th International Workshop on Lessons from Animal Diabetes

**DOI:** 10.1155/EDR.2001.251

**Published:** 2001

**Authors:** Palle Serup, Jan Jensen, Jacob Hald, Ole D. Madsen

**Affiliations:** Department of Developmental Biology Hagedorn Research Institute Gentofte Denmark


*Int. Jnl. Experimental Diab. Res.*, Vol. 2, pp. 251–297
Copyright © 2001 Taylor & Francis, Inc.
1560-4284/01 $12.00 + .00

### Abstracts of the 8th International Workshop on Lessons from Animal Diabetes

Joint with
The 15th Japan Association of Animal Diabetes Research

TOKYO, JAPAN, JULY 24–26, 2001

### Pancreatic Stem Cells and Islet Cell Differentiation

PALLE SERUP, JAN JENSEN, JACOB HALD and OLE D. MADSEN

*Department of Developmental Biology, Hagedorn Research Institute, Gentofte, Denmark*

During the embryonic growth mechanisms that are not fully clear ensures that exo- and endocrine cells are formed in the correct proportion. The embryonic endocrine progenitor cells are a subset of the developing $Pdx1^+$ pancreatic epithelial cells marked by the expression of *Neurogenin3* (*Ngn3*). *Ngn3* encodes a basic-Helix-Loop-Helix (bHLH) transcription factor (NGN3) that is required for the expression of *NeuroD* (as well as other transcription factor genes); moreover, ectopic *Ngn3* expression can induce differentiation of embryonic pancreatic epithelium into α-cells at the expense of other pancreatic cell types. Notch signaling appears to control the activity of NGN3 and acts as a switch that determines the choice of the progenitor cells; the choice being to either remain as dividing precursor cells or to differentiate into endocrine cells. NGN3 is thought to activate transcription of the Notch ligand Dll–1. Once Dll–1 expression is induced in a differentiating cell it interacts with Notch, expressed on neighbouring cells. Notch activates a number of genes among which is the negatively acting bHLH gene *Hes-1*. This pathway (termed lateral inhibition) thus inhibits NGN3 activity and consequently endocrine differentiation and *Dll–1* expression in the receiving cells. Lateral inhibition assures that only a few precursor cells differentiate and that a large fraction of the endocrine precursor cells in the pancreas are maintained in a dividing state until late stages of pancreatic development. In mice deficient for Notch pathway components, the precursor cells differentiate prematurely into endocrine cells. (Supported by NIH DK-55284)

### Molecular Pathogenesis of Type 2 Diabetes in Knockout Mice Models

TAKASHI KADOWAKI, M.D., Ph.D.

*Department of Metabolic Diseases, Graduate School of Medicine, University of Tokyo*

Type 2 diabetes is a complex disease caused by interactions of multiple genes and environmental factors such as high-fat diet and sedentary life-style. Since type 2 diabetes is characterized by insulin resistance and relative insulin deficiency, we have tried to dissect molecular pathogenesis of type 2 diabetes by generating several knockout mice models with a lack of each key molecules of signalling pathways of either insulin action or insulin secretion such as insulin receptor substrate-1 (IRS-1),[1] IRS-2,[2] PI3 kinase,[3] PPARg,[4] b-cell glucokinase,[5] or the NADH shuttle system.[6] Moreover, in order to study gene-gene and gene-environment interactions in the development of type 2 diabetes, we have crossed these animals [7] and/or exposed them to a certain environmental factors such as high-fat diet.[4] DNA chip analysis has also been used to systematically identify crucial pathways for the development of type 2 diabetes in each tissue of these animals.

These efforts have begun to reveal some key aspects of molecular pathogenesis of type 2 diabetes.[8] We have found that insulin resistance can lead to type 2 diabetes only when b cell defects exist simultaneously such as decreased insulin response to glucose [7] or impaired b cell hyperplasia.[2] We have also found that PPARg plays a crucial role in the development of high-fat diet-induced obesity, insulin resistance and type 2 diabetes.[4]

Importantly, pathways identified to have a role in the development of type 2 diabetes in these analyses can be tested as candidate susceptibility loci of human type 2 diabetes. Conversely, functional impact of human type 2 diabetes susceptibility loci can be analyzed by generating corresponding mutant mice. Finally, we have begun to apply information obtained in these analyses to develop potential tailar-made medicine to provide fundamental prevention and therapeutic strategies for type 2 diabetes.[9]

### *References*

[1] Tamemoto, H. *et al.* (1994). *Nature*, **372**, 182–186.
[2] Kubota, N. *et al.* (2000). *Diabetes*, **49**, 1880–1889.
[3] Terauchi, Y. *et al.* (1999). *Nature Genetics*, **21**, 230–235.
[4] Kubota, N. *et al.* (1999). *Mol. Cell*, **4**, 597–609.
[5] Terauchi, Y. *et al.* (1995). *J. Biol. Chem.*, **270**, 30253–30256.
[6] Eto, K. *et al.* (1999). *Science*, **283**, 981–985.
[7] Terauchi, Y. *et al.* (1997). *J. Clin. Invest.*, **99**, 861–866.
[8] Kadowaki, T. *et al.* (2000). *J. Clin. Invest.*, **106**, 459–465.
[9] Kato, H. *et al.* (2001). *Diabetes*, **50**, 113–121.

### C-Peptide Deficiency—A Pathogenetic Factor in Type 1 Diabetic Complications

ANDERS A. F. SIMA

*Wayne State University, Detroit, MI, USA*

In recent years the physiological role of proinsulin C-peptide has received increasing attention, focusing on its potential role in preventing and ameliorating type 1 diabetic complications. Studies suggest an interaction between C-peptide and insulin. In human skeletal muscle strips, C-peptide stimulates glucose transport and in rat L6 myoblasts, physiological concentrations of C-peptide autophosphorylates the insulin receptor, stimulates tyrosine kinase, IRS-1 tyrosine phosphorylations, P13 kinase activity and MAP kinase phosphorylation. These effects result in stimulated glycogen synthesis and amino acid uptake. These data suggest that the insulin signaling pathway may be activated by both C-peptide and insulin, although C-peptide does not compete with insulin binding. In type 1 diabetic patients C-peptide improves heart rate variability and temperature threshold discrimination. In type 1 diabetic neuropathy in the BB/Wor-rat C-peptide replacement prevents and ameliorates the acute $Na^+/K^+$-ATPase defect and nerve conduction deficits. Chronic C-peptide replacement or intervention prevents and partially reverses nerve conduction velocity, axonal atrophy, myelinated fiber loss and the nodal structural changes characteristic of type 1 diabetic neuropathy. The latter effects are probably associated with improved insulin activity via co-localized insulin receptors at the paranodal apparatus. Additional mechanisms involved in these effects include its stimulating effect on eNOS resulting in increased NO release. In summary, experimental and clinical data convincingly demonstrate that C-peptide exerts biological effects, which appears to be mediated by its insulinomimetic effects. These effects translate into preventive and therapeutic effects on metabolic, functional and structural abnormalities in type 1 diabetic complications.

### Diabetic Neuropathy: Lessons from Animal Models

MARTIN STEVENS, M.D.

*University of Michigan Medical Center*

Diabetic peripheral neuropathy (DPN) is a complex multifactorial disorder, characterized by nerve fiber atrophy and loss. Experimental DPN is marked by impaired nerve conduction velocity (NCV), reduced nerve blood flow, nerve energy deficits and a variety of metabolic abnormalities in peripheral nerve, that have been variously ascribed to hyperglycemia, abnormal fatty acid metabolism, ischemic hypoxia, and / or oxidative stress. The nature of underlying metabolic and / or vascular insults and their precise cellular localization remain highly speculative. It is apparent that the multiple insults resulting from glucose toxicity eventually summate to result in nerve degeneration and regeneration, the balance of which determines the subsequent fate of the nerve. One of the best studied metabolic deficits implicated in the development of DPN has been glucose induced activation of the polyol pathway. This results in wide-ranging metabolic and vascular deficits, many of which have been invoked in their own right, as being pivotal in the development of DPN. Recently, oxidative stress has emerged as a critical factor in the development of DPN. Hyperglycemia promotes oxidative stress through both non-enzymatic and enzymatic mechanisms. Non-enzymatic protein glycation is thought to generate reactive oxygen species (ROS) though a complex series of chemical and cellular intermediates. ROS also have direct neurotoxic activity, promoting neuronal apoptosis and mediating ischemia-reperfusion injury in the nervous system. Conversely, neurotrophic factors protect neurons against oxidative stress and upregulate antioxidative defense mechanisms. Thus, the interrelationships between hyperglycemia, polyol pathway, oxidative stress, endoneurial hypoxia, nerve energy depletion, impaired neurotrophic support and slowed NCV in experimental DPN may be complex, and may reflect the heterogeneous and compartmentalized composition of peripheral nerve. Recently, the use of selective pharmacological interventions has demonstrated the interdependence of many of the glucose-induced metabolic and vascular deficits, and that therapeutic synergism exists between these different pathways. Understanding the mechanisms by which hyperglycemia results in neuropathy is critical for the development of new therapeutic interventions aimed at attenuating degenerative processes and enhancing nerve regeneration.

### Prevention of Nephropathy: Lessons from Animal Models

MASAKAZU HANEDA, DAISUKE KOYA and RYUICHI KIKKAWA

*Third Department of Medicine, Shiga University of Medical Science, Otsu, Shiga, Japan*

Recent reports suggest that the activation of diacylglycerol (DAG)-protein kinase C (PKC)-extracellular signal-regulated kinase (ERK) pathway under diabetic conditions plays a key role in the development of diabetic nephropathy. To prove this hypothesis, we examined whether the inhibition of DAG-PKC-ERK pathway could prevent the development of glomerular dysfunction in diabetic animals. Since we found that thiazolidinedione compounds could inhibit PKC activation by activating DAG kinase which converted DAG to phosphatidic acid, we examined the effect of troglitazone and pioglitazone on early glomerular dysfunction in streptozotocin-induced diabetic rats. Thiazolidinedione compounds were able to prevent glomerular hyperfiltration, albuminuria, and excessive production of extracellular matrix (ECM) proteins in glomeruli of STZ-induced diabetic rats without changing plasma glucose levels. Therefore, STZ-induced diabetic rats are considered to be a useful model for evaluating early glomerular dysfunction in diabetes. However, it is difficult to evaluate glomerular histological changes in STZ-induced diabetic rats. For this purpose, we use db /db mice, a model for type 2 diabetes, because these mice were found to develop significant mesangial expansion at 16 weeks of age. In db /db mice, we tried to inhibit PKC directly by an oral administration of PKC $\beta$ inhibitor. PKC $\beta$ inhibitor was given for 16 weeks from 9 weeks of age and glomerular histological changes were evaluated. Fractional mesangial area and the expression of

ECM proteins in mesangium were significantly increased in glomeruli of db/db mice as compared with db/m mice. PKC β inhibitor was able to prevent these glomerular histological changes in db/db mice together with a significant reduction of albuminuria. These results indicate that different animal models for diabetes can be used for the evaluation of different glomerular changes and the results confirmed the importance of the activation of DAG-PKC-ERK pathway in the development of glomerular dysfunction in diabetes.

### A New Rat Model of Diabetic Retinopathy

AKIHIRO KAKEHASHI

*Department of Ophthalmology, Omiya Medical Center, Jichi Medical School, Saitama, Omiya, Japan*

I report here details of diabetic ocular complications observed in a new model of spontaneously diabetes in non-obese Torii rats (SDT rat). The SDT rat is a sub-strain of the Sprague-Dawley (SD) rat, which spontaneously develops diabetes mellitus. The male SDT rats develop marked hyperglycemia (about 700 mg/dl) after 20 weeks of age, and the female rats develop hyperglycemia after 45 weeks of age. The SDT rats have common features of diabetic ocular complications such as cataract, diabetic retinopathy, and neovascular glaucoma. 1) A mature diabetic cataract developed before 40 weeks of age. 2) Diabetic retinopathy developed after 55 weeks of age in most cases. A diabetic tractional retinal detachment and vascular abnormality were observed frequently, but retinal hemorrhage did not occur as frequently. Fluorescein angiography shows hyperfluorescence, suggesting retinal neovascularization. Acellular capillaries with pericyte loss and extensive narrowing of retinal vessels were observed in most cases using the trypsin digestion method. Electron microscopy showed the thickened basement membrane of the retinal vessels. 3) Neovascular glaucoma: a neovascular fibrous membrane around the iris and a massive hemorrhage were observed in the anterior chamber in advanced cases. The ocular complications of this rat mimic those found in diabetic patients. The SDT rat model may be the first animal model with spontaneously occurring proliferative diabetic retinopathy. I believe that this rat model is extremely useful for studying pathogenesis and the treatment of diabetic ocular complications.

### Alteration of Vasoactive Factors in Chromic Diabetic Complication

DR. SUBRATA CHAKRABARTI

*The University of Western Ontario, Department of Pathology, London, Ontario, Canada*

Vasoactive factors like endothelins (ETs) by virtue of their widespread action are of significant importance in the pathogenesis of several diabetic complications. We investigated streptozotocin induced diabetic rats and galactose fed rats after one and six months of diabetes with respect to ETs and other interactive molecules.

Both diabetes and galactosemia upregulated components of ET system in the retina and kidney. Diabetes further caused similar changes in the heart after one month of follow-up. ET receptor blockade prevented hyperhexosemia induced albuminuria and increased resistivity index (a marker of peripheral vasoconstriction) of the retina. After six month of follow-up hyperhexosemia induced augmented ECM protein synthesis and increased capillary basement membrane thickening in the retina and kidney were prevented by a dual $ET_A/ET_B$ receptor antagonist. Diabetes further caused focal myocardial fibrosis in the heart which was also prevented by ET receptor antagonism. In diabetes increased VEGF mRNA expression and reduced iNOS mRNA expression were observed in retina and heart. In the endothelial cells, retina and heart, ET-receptor blockade normalized augmented VEGF mRNA and reduced iNOS mRNA expression. VEGF blockade also reduced diabetes induced increased ET mRNA expression and permeability in the retina.

Data from these studies suggest that ETs by themselves and by their interaction with other vasoactive factors play an important role in the pathogenesis of chronic diabetic complications. Blockade of some of these pathways may represent potential adjuvant therapies for the treatment of chronic diabetic complications. [Supported in part by grants from CDA & CIHR]

### Mechanisms of the Prevention of Diabetes by Mitigation of Obesity

BARBARA CALEEN HANSEN

*University of Maryland School of Medicine, Baltimore, MD, USA*

Primary prevention of type 2 diabetes mellitus can be achieved by prevention of obesity in monkeys (Diabetes 42:1809, 1993). In humans, prevention of type 2 diabetes, or at least delay of its onset, has been associated with changes in life style which include reduction in body weight, total fat intake, and saturated fat, and increased in fiber intake and physical activity (N Engl J Med 344:1343, 2001), although which of these factors carries the power to mitigate diabetes risk is unknown. Evidence suggests that type 2 diabetes is the result of an interaction of genetic factors and a permissive environment, however, only the amount of body fat was manipulated in the non human primate study, with no change in activity or diet composition. We have, therefore, focused upon the roles of reduced calorie flux (calorie intake restriction and reduced calorie output per lean body mass) and its major consequence, reduced the adipose tissue, in postponing or preventing the development of overt diabetes. During long term modest calorie restriction, sufficient to sustain the normal adult body composition of a 10 year old monkey ($X$ = 22% body fat, range 18.7 to 25.7%) the percent body fat of total body weight increased by only 1.8%. This is despite the fact that these monkeys are approaching 30 years of age. Plasma leptin levels did not change with continued maintenance of stable weight. Although overnight fasted skeletal muscle glycogen levels were unchanged by long term calorie restriction, the glycogen storage pathway appeared to be upregulated. Since energy expenditure is down regulated, we suspect a reduction in futile cycling in several metabolic pathways. These metabolic alterations may be involved in the mechanisms of the health promoting effects of calorie restriction and of calorie restriction-mimetic agents.

### Physiology of Fatless Mice

OKSANA GAVRILOVA, LILY CHAO and MARC L. REITMAN

*Diabetes Branch, National Institute of Diabetes and Digestive and Kidney Diseases, National Institutes of Health, Bethesda, MD 20892-1770, USA*

We have generated a transgenic mouse (A-ZIP/F-1) virtually lacking white adipose tissue (WAT). The A-ZIP/F-1 phenotype resembles that of humans with severe lipoatrophic diabetes, including the lack of fat, marked insulin resistance and hyperglycemia, hyperlipidemia, and fatty liver. Lipoatrophic diabetes is in striking contrast to the usual association of diabetes with obesity. We reversed the metabolic phenotype by surgical transplantation of adipose tissue. This demonstrated that the metabolic derangements associated with lipoatrophy are actually caused by the lack of adipose tissue. Leptin infusion was only modestly effective in treatment of the diabetes, suggesting that leptin deficiency contributes, but may not be the sole mechanism by which WAT prevents diabetes.

There is uncertainty about the sites of action of the antidiabetic thiazolidinediones (TZDs). TZDs are agonist ligands of the transcription factor PPARγ, which is abundant in adipose tissue, but is normally present at very low levels in liver and muscle. In A-ZIP/F-1 mice, rosiglitazone or troglitazone treatment did not reduce glucose or insulin levels, suggesting that white adipose tissue is required for the antidiabetic effects of TZDs. However, TZD treatment was effective in lowering circulating triglycerides and increasing whole body fatty acid oxidation in the A-ZIP/F-1 mice, indicating that this effect occurs via targets other than white adipose tissue.

Rosiglitazone was also observed to have actions on steatotic livers that it does not have in control livers, such as increasing triglyceride content.

### Transgenic Skinny Mice Overexpressing Leptin

YOSHIHIRO OGAWA and KAZUWA NAKAO

*Department of Medicine and Clinical Science, Kyoto University Graduate School of Medicine, Kyoto, Japan*

Leptin is an adipocyte-derived blood-borne satiety factor that plays a role in a variety of homeostatic processes. Plasma leptin concentrations are elevated in obese subjects in proportion to the degree of adiposity, suggesting the state of leptin resistance in obese subjects. On the other hand, it is conceivable that hyperleptinemia may play a role in the pathogenesis of obesity and obesity-related disorders. To explore the long-term effect of leptin *in vivo*, we created transgenic skinny mice with elevated plasma leptin concentrations comparable to those in obese subjects. Chronic overexpression of leptin in the liver has resulted in complete disappearance of white and brown adipose tissue for a long period of time. We call these animals transgenic skinny mice. Transgenic skinny mice exhibit increased glucose metabolism and insulin sensitivity compared to nontransgenic littermates accompanied by the activation of insulin signaling in the skeletal muscle and liver. They also show blood pressure elevation compared to nontransgenic littlermates through the activation of sympathetic nervous system. Furthermore, female transgenic skinny mice exhibit accelerated puberty and late-onset hypothalamic hypogonadism. These observations indicate that our transgenic skinny mice will serve as a new genetic model with chronic hyperleptinemia and suggest the pathophysiologic and therapeutic implication of leptin in obesity and obesity-related disorders.

### Characteristics and Limitations of the Wistar Fatty Rat Strain as an Obese-Diabetic Model

HITOSHI IKEDA

*Pharmaceutical Research Division, Takeda Chemical Ind., Ltd., Osaka, Japan*

Wistar fatty (*fa/fa*, WF) rate strain was established by transferring the *fa*-gene from the Zucker rat (13M strain) to the Wistar Kyoto (WKY) rat strain that has less sensitivity to insulin and less tolerance to glucose. After the 5$^{th}$ generation of backcrossing, male obese hybrid rats were found to be hyperglycemic. This finding clearly demonstrated a close relationship between genetic and environmental factors in the diabetogenic action of obesity. Thereafter, WF rats were confirmed to be a good model for human type 2 diabetes mellitus with hepatic and peripheral insulin resistance, and used to evaluate efficacy and the mechanism of insulin sensitizers. The recent findings raised the question of whether the WF rat strain is a good model for human obesity. It has been recognized that leptin and leptin receptors play key roles in the regulation of body weight through the regulation of feeding behavior and energy expenditure. Although a high concentration of plasma leptin levels and/or leptin resistance are common phenomena in both human and animal obesity, the causes responsible for them seem to be quite different. The well-known obese rodents, such as *ob/ob* mice or *db/db* mice and *fa/fa* rats, have mutations in leptin or its receptors. On the contrary, only very rare cases of human obesity have such mutations. Therefore, the WF rat strain as well as other animal models with a single gene-mutation, can possibly mislead the investigation and/or development of new drugs in the field of feeding regulation. What is the most suitable model for human obesity?

### Present Status of Genetic Analyses of Causative Genes on the Diabetic Strains of Mouse and Rat Established in Japan

KAJURO KOMEDA[a], MITSUHIKO NODA[b] and YASUNORI KANAZAWA[c]

*[a]Tokyo Medical University, Tokyo, [b]Institute for Diabetes Care and Research, Asahi Life Foundation, Tokyo,*
*[c]Omiya Medical Center, Jichi Medical School, Omiya, Japan*

Human diabetes mellitus is a disease characterized by a complex phenotype caused by environmental factors in subjects having a diabetogenic gene(s), which is usually polygenic. Because restricted information obtained from human beings is limited due to ethical reason particularly for genetic analyses, spontaneously diabetic animal models of human diabetes are important in identifying these genes in human beings. In Japan, many polygenic models of mouse and rat have been established, such as KK, NOD, NSY and TSOD mice, and GK, OLETF, KDP and SDT rats. These strains show a pathophysiological phenotype similar to diabetes in humans, and considerable effort has been exerted thus far to identify the genes underlying susceptibility to diabetes in these animals. From these viewpoints, the following will be discussed in this presentation: 1) overview of the characteristics of the polygenic animal models for human diabetes; 2) a new "causative gene animal model" derived from pathophysiological phenotype animal models; 3) genetic mapping of susceptibility loci in the polygenic animal models; 4) recent ideas on genetic analyses of the polygenic animal models by the positional candidate gene approach after genetic mapping of the diabetogenic loci: the use of chromosome-substitution strains, importance of establishment of congenic strains, and requirement of the mouse/rat/human comparative genetic map; and 5) possible advances in this field in the near future.

### Susceptibility to Diabetes Is Widely Distributed in Normal Class II$^U$ Haplotype Rats

K. E. ELLERMAN and A. A. LIKE

*Department of Pathology, University of Massachusetts Medical School, Worcester, MA, USA*

*Aims/hypothesis.* We did experiments to explore the pathways putatively leading to Type I (insulin-dependent) diabetes mellitus, and their association with the MHC locus, the major genetic determinant of disease susceptibility.

*Methods.* Normal MHC congenic rat strains that do not spontaneously develop diabetes or any other autoimmune syndrome were injected with the interferon-alpha inducer polyinosinic-polycytidylic acid (Poly IC).

*Results.* Insulitis and diabetes developed only in strains expressing Class II$^u$ genes and was independent of the Class I haplotype. Poly IC induced islet cell Class I hyperexpression, up regulation of pancreatic endothelial intercellular adhesion molecule-1 and vascular adhesion molecule-1 and a T-cell and macrophage infiltration of the pancreatic interstitium in all rat strains studied, including diabetes-resistant strains. Poly IC also induced the generation of diabetes-transferring spleen cells in most Class II$^u$ haplo-type rats, including the diabetes-resistant *WF* rat.

*Conclusion/Interpretation.* The minimum requirements for autoimmune diabetes development in the rat include: *RT1*Class II$^u$ genes, a T-cell repertoire containing beta-cell autoreactive T cells and a triggering event which breaks tolerance by the local up regulation of pancreatic endothelial adhesion receptors. Even when all of the minimum requirements have, however, been met, most Class II$^u$ rats do not develop diabetes in response to autoimmune stimuli. It is clear, nonetheless, that susceptibility to diabetes is widely distributed in the *RT*$^u$ rat.

### Rodent Models in Diabetes Research: Value Enhanced Through Sharing

EDWARD H. LEITER

*The Jackson Laboratory, Bar Harbor, ME 04609*

The Jackson Laboratory did not begin its existence as a supplier of mice. As the research staff found interesting mutants, it became part of the institutional "culture" to distribute the mice to all interested investigators. Over time, this necessitated creation of a distribution department to accommodate the many requests. This "culture" of facilitating rapid, widespread distribution of new mouse models of human disease is now the norm among most of the international research community involved in generating new rodent models of research into diabetes and its complications. The success of Japanese investigators in developing new rat and mouse models for diabetes research is viewed with justifiable pride by the Japanese research community. However, in the case of some of these models, a reluctance to allow international distribution have limited scientific inquiry to the detriment of diabetes research. The purpose of this presentation will be to highlight how the scientific value of any model developed in Japan is greatly enhanced by promoting and facilitating its widespread international distribution. Two recent models recently sent by Japanese investigators to be studied world-wide will be highlighted; the C57BL/6-*Ins2*$^{Akita}$ mutant mouse and the ALS/ALR inbred strains.

### A Significance of Adipose Tissue within OLETF Rats

TSUKASA HIRASHIMA[a], YANG LUAN[b], ZHI-WEI MAN[c] and KAZUYA KAWANO[a]

[a]*Department of Research Management, Otsuka Pharmaceutical Co., Ltd., Tokushima, Japan,* [b]*Shenyang Pharmaceutical University, Shenyang, China,* [c]*First Institute of New Drug Research, Otsuka Pharmaceutical Co., Ltd., Tokushima, Japan*

It is known that OLETF rats reveal hyperphagia, hypertriglyceridemia and obesity, and these symptoms are related to development of diabetes. However, pathogenesis of hypertriglyceridemia and obesity has not yet been fully elucidated. In order to eliminate the effects of hyperphagia, OLETF rats, subjected to restricted feeding from 5 to 21 weeks of age (R-OLETF), were given the same amount of food daily as LETO rats, which were used as control. There was no significant difference in body weight at 20 weeks of age between R-OLETF and LETO rats. However, sum of plasma glucose in OGTT, fasting plasma glucose, insulin, triglyceride (TG),and leptin levels in R-OLETF rats were significantly higher than in LETO rats. Moreover, the weight of body fat tissues and the rate of body fat in R-OLETF rats were also significantly higher than in LETO rats. Activity of monoacylglycerol acyltransferase (MGAT), which plays an important role in TG synthesis, was significantly higher in the small intestine and liver of R-OLETF rats than in LETO rats. These results indicate that triglyceride synthesis in OLETF rats was accelerated, although OLETF rats were subjected to restricted feeding, and this may be involved in the pathogenesis of hypertriglyceridemia, body fat accumulation, and development of diabetes in OLETF rats.

### Are Rodent Models Predictive of Diabetic Neuropathy in Man: Case Study for Aldose Reductase Inhibitors

THOMAS C. HOHMAN

*Pharmacia Corporation, Peapack, NJ, USA*

In contrast to their efficacy in animal studies, Aldose Reductase (AR) Inhibitor treatment in diabetic patients has produced little evidence of clinical benefit. Dose ranging studies in experimental models of diabetic neuropathy suggest that the clinical benefit of ARIs may have been limited by the testing of doses that caused less than complete inhibition of target tissue AR. In diabetic animals, ponalrestat, sorbinil and tolrestat, tested at doses that completely inhibited sorbitol accumulation in the sciatic nerve prevented or reversed the development of neural lesions. At the highest dose tested in clinical studies, ponalrestat had to effect on sorbitor levels in human sural nerve, while sorbinil and tolrestat reduced sorbitol levels by 42 and 60%, respectively. For all three compounds, the biochemical results were accompanied by occasional improvements in nerve conduction velocity (NCV) that did not persist. In more recent clinical studies with zenarestat and minalrestat where sural nerve sorbitol was suppressed by 85 to 90%, NCV was improved by 1 to 3 m/s. For zenarestat an improvement of NCV was observed in 3 out of 4 nerves. In spite of the apparent relationship between sural nerve sorbitol levels and an improvement in NCV, tissue polyol levels may be poor predictors of functional effects. In animal studies where AR inhibitor treatment was withdrawn, the benefits of treatment disappeared with 2 to 4 days, while nerve sorbitol inhibition persisted for 10 to 12 days. These results suggest that tissue polyol levels may not provide accurate estimates of the level of AR inhibition or flux through the polyol pathway. Alternatively, the results may suggest that the metabolic imbalance in the nerve due to increased flux through the polyol pathway is not the primary target for AR inhibitors.

### Antioxidant Treatment of Streptozotocin-Induced Diabetic Rats: Effect on Endoneurial Blood Flow (EBF), Motor Nerve Conduction Velocity (MNCV), and Endothelial-Dependent Vascular Relaxation of Epineurial Arterioles of the Sciatic Nerve

MARK A. YOREK

*University of Iowa, Iowa City, IA, USA*

We have shown that diabetes-induced reduction in EBF and impaired endothelium-dependent vascular relaxation of arterioles that provide circulation to the region of the sciatic nerve precedes slowing of MNCV. Furthermore, vascular dysfunction was accompanied by an accumulation of superoxide and peroxynitrite in epineurial vessels. The purpose of this study was to examine whether treatment of diabetic rats with antioxidants can prevent the generation of superoxide as well as diabetes-induced vascular and neural dysfunction. Diabetic rats were treated with 0.5% a-lipoic acid (LA) (a free radical scavenger and transition metal chelator), 10 mg/kg M40403 (a non-peptidyl mimic of superoxide dismutase) or 75 mg/kg hydroxyethyl starch deferoxamine (HES-DFO) (a transition metal chelator) for 3–4 weeks. Following treatment we found that each of these antioxidants improved the diabetes-induced decrease in EBF, vascular relaxation of epineurial vessels and MNCV. The treatments also reduced the production of superoxide and peroxynitrite by epineurial vessels. Treatment with LA and M40403 but not HES-DFO prevented the diabetes-induced increase in serum thiobarbituric acid reactive substances. Treatment with LA but not M40403 or HES-DFO partially improved sciatic nerve $Na^+/K^+$ATPase activity. These studies suggest that oxidative stress caused by the diabetes-induced generation of superoxide and/or peroyxnitrite in epineurial vessels may be partially responsible for the development of diabetic vascular and neural complications.

### Polyol-Age Interplay in Diabetic Complications: Clue from Animal Models

SOROKU YAGIHASHI, SHIN-ICHIRO YAMAGISHI, RUICHI WADA and CHIEKO HANYU

*Department of Pathology, Hirosaki University School of Medicine, Hirosaki, Japan*

Long-standing hyperglycemia has been implicated in the pathogenesis of chronic complications in diabetes. Excessive flux of polyol metabolism, increased non-enzymatic glycation, overproduction of reactive oxygen species (ROS) and increased protein kinase C (PKC) activity are all considered to attribute to the development of diabetic complications. Recent clinical and experimental data suggest that these abnormalities may at least in part take a common process for the development of characteristic pathology. However, it is not known as to the precise mechanisms of how these metabolic aberrations lead to cellular injuries. The differences in the lesions between polyol- and glycation-associated changes are not clear. Attempts to inhibit the development of neuropathic changes with aldose reductase inhibitors (ARI) or with aminoguanidine (AG), anti-glycation agent, have disclosed some differential effects on the neuropathic changes in diabetic animal models. Using transgenic mice overexpressing aldose reductase, ARI could be only beneficial for diabetic mice with high AR contents, whereas AG only partially improved neuro-vascular dysfunction in streptozotocin-induced diabetic rats. PKC activities were differently altered between endoneurium and vessel-rich peri-neurial tissues in diabetic animals. ROS-induced cellular injury occurs in nerve fibers via AGE-induced vascular injury as well as through in situ accumulation of AGE. These findings indicate that the prevention and treatment of chronic neuro-vascular complications should be explored from multifaceted aspects of their pathogenesis. (Supported in part by Japanese Ministry of Education, Science, Culture, and Sports and Juvenile Diabetes Research Foundation)

### Autoantibody against $N^{\varepsilon}$-(Carboxymethyl)lysine as a Potential Maker for Diabetic Nephropathy and Chronic Renal Failure

SEIKOH HORIUCHI, RYOJI NAGAI, WAKAKO KOITO and TAMAMI SAKAMOTO

*Department of Biochemistry, Kumamoto University School of Medicine, Kumamoto, Japan*

$N^{\varepsilon}$-Carboxymethyl)lysine (CML) is one of the major structures of advanced glycation end products (AGE). Extensive immunological studies using anti-CML antibodies have demonstrated the presence of CML-protein adducts in several human and animal tissues, indicating a potential link of CML-modification to aging and age-enhanced disease processes such as diabetic complications. In the present study, we tested whether CML-structures present *in vivo* could serve as immunogens to generate autoantibodies using plasma and tissue samples from rats and humans. First, the anti-CML antibody (6D12) reacted positively with extracts of erythrocytes, lens, kidney, aorta, and sciatic nerve from STZ-induced diabetic rats. Plasma IgG of these animals reacted with AGE-BSA, but not with BSA. The reactivity was increased with the duration of diabetic states and inhibited specifically by CML-BSA. Secondly, a fraction was purified from plasma of diabetic patients which bound to AGE-BSA but not to BSA. Finally, plasmas from patients of five different categories (diabetes without renal failure, diabetes with microproteinuria, diabetes with macroproteinuria, diabetes with hemodialysis and non-diabetes without hemodialysis) were compared with those of normal subjects in the autoantibody activity against CML. Results showed that patients with renal failure caused by diabetic or non-diabetic pathologies had a higher autoantibody activity than in normal subjects or diabetic patients without renal failure. These findings indicate that CML accumulated *in vivo* serves as an immunological epitope to generate a CML-specific autoantibody which might be used as a potential marker for diabetic nephropathy or chronic renal failure.

### Emerging Therapies for Insulin Resistance: Lessons from Animal Models

JULIE S. MOYERS and JOSEPH T. BROZINICK

*Endocrine Research, Eli Lilly and Company, Indianapolis, IN, USA*

An understanding of the regulation of insulin action at the molecular and cellular level is key to discovering the mechanisms of insulin resistance that underlie type 2 diabetes. The translation of that knowledge into rodent models of insulin resistance and diabetes has been a powerful approach to validate some of the numerous signaling molecules that could serve as targets for therapies to treat insulin resistance in humans. Emerging evidence suggests that targets at several levels in the insulin signaling cascade may serve as viable sites for therapies. While the possibility of identifying compounds other than insulin that directly activate the insulin receptor has long appeared remote, a non-peptide small molecule has recently been identified that activates the insulin receptor in *in vitro* and *in vivo* (Zhang *et al.*, 1999). Additional progress has been made in targeting inhibitory phosphatases where small molecule PTP1B inhibitors have been identified that exert glucose lowering effects *in vivo* (Wrobel *et al.*, 1999). Further downstream in the insulin signaling cascade, GSK-3 activity was found to be elevated and insensitive to inhibition by insulin in isolated muscle from ZDF rats and obese insulin-resistant humans, while expression levels remained unchanged. Additionally, treatment of 8 week old ZDF diabetic animals with lithium, an inhibitor of GSK-3, blunted glucose elevation in this animal model. Associated with this, incubation of isolated epitrochlearis muscles from these rats with lithium enhanced GSK-3 phosphorylation, glycogen synthase activity, glycogen synthesis and glucose transport. Thus, while we are faced with the difficult challenge to design therapeutic strategies to treat insulin resistance, several emerging approaches and lessons from animal models offer promise.

### Mechanisms behind Impaired Insulin Release in the Spontaneously Diabetic Goto-Kakizaki (GK) Rat

CLAES-GORAN OSTENSON

*Department of Molecular Medicine, Endocrine & Diabetes Unit, Karolinska Hospital and Institute, Stockholm, Sweden*

The GK rat is a non-obese substrain of Wistar rat origin, developing type 2 diabetes-like syndrome early in life. Impaired B-cell function plays an instrumental role for glucose intolerance against a polygenic diabetic inheritance and environmental influences, *e.g.*, gluco- and lipotoxicity. Defective insulin response to glucose may be due to impaired ATP generation and thereby improper closure of K-ATP channels in B-cells. In GK rat islets, we have demonstrated several abnormalities that may have such effect, *i.e.*, increased glucose cycling and glucose-6-phosphatase activity, decreased glycerol phosphate dehydrogenase activity and shuttle, and decreased pyruvate dehydrogenase and carboxylase activities. However, all these abnormalities were normalised after restoring glycemia *in vivo* by insulin or phlorizin treatment, suggesting glucotoxocity. In addition, decreased islet exocytotic SNARE complex proteins (syntaxin-1A, SNAP-25, VAMP-2 and nSec1) in GK rat islets were nearly normalised after normalisation of glycemia, suggesting yet another mechanism for glucotoxic impairment of insulin release.

Reduced ATP levels in B-cells might also be due to increase islet phosphatase activity. Indeed, in GK rat islets mRNA levels and protein amounts of the protein tyrosine phosphatase (PTP) NE-3 have been shown increased, by about 60%, and unrelated to hyperglycemia. Treatment *in vitro* with antisense to the phosphatase decreased the protein amounts in parallel to improvement of the insulin response to glucose. Overexpression of PTP NE-3 in B-cells may thus be a primary factor behind the impaired insulin release in the GK rat.

### Pathophysiology of GK Rats

YUTAKA SEINO

*Department of Metabolism and Clinical Nutrition Graduate School of Medicine, Kyoto University, 54 Shogoinkawahara-cho, Sakyo-ku, Kyoto, Japan*

In the Goto-Kakizaki rat, a genetic model of type 2 diabetes, in addition to decreased insulin synthesis, insulin response to glucose is selectively impaired. To elucidate the mechanism of this abnormality, we studied the properties of ATP-sensitive $K^+$ channels, the inhibition of which is a key step of insulin secretion induced by fuel substrates, using the patch-clamp technique. The glucose sensitivity of KATP channels was considerably reduced in GK rats. However, the inhibitory effects of ATP on channel activity and unitary conductance were not significantly different between control and GK rats. Thus, it appears that the impaired insulinotropic action of glucose in β-cells of GK rats is attributable to insufficient closure of the $K^+$ channels, probably because of deficient ATP production by impaired glucose metabolism. $K_{ATP}$-channel activities in both control and diabetic β-cells were found to be equally suppressed by glyceraldehyde and 2-ketoisocaproate. These results strongly suggest that the step responsible for the metabolic dysfunction of the diabetic β-cells is located within the glycolytic pathway before glyceraldehyde-3-phosphate or in the glycerol phosphate shuttle.

To clarify whether or not impaired insulin gene transcription exists in GK rats, we investigated the expression and function of the transcriptional repressor CREM (CRE modulator) in pancreatic islets. The CREM gene generates both transcriptional activators and repressors by alternative splicing and an intronic promoter. We isolated a novel alternatively spliced CREM isoform, CRED-17X, which efficiently represses insulin gene transcription, in addition to the three previously reported repressors. We also compared mRNA levels of insulin and the CREM repressors in pancreatic islets of Wistar and GK (Goto-Kakizaki) rats. The CREM repressor levels are increased, and the expression of insulin mRNA is decreased in GK rats, suggesting that increased CREM repressor expression in pancreatic islets could contribute to the decreased insulin gene transcription that results in impaired insulin secretion in GK rats.

### GK Rats

KEN-ICHI SUZUKI and YOSHIO GOTO

*Department of Metabolism and Diabetes Mellitus, Tohoku Kosei-nenkin Hospital, Sendai, Miyagi, Japan*

Impaired insulin response, insulin resistance or both characterize type 2 diabetes mellitus. GK rat is a non-obese, mildly diabetic rat that was developed by inbreeding Wistar rats with selection of rats in each generation with the highest blood glucose levels during an oral glucose tolerance test. The insulin secretion stimulated by glucose is markedly impaired in GK rats. In perifusion, glucose-stimulated insulin release was reduced by 90% for first phase and by 75% for second phase. The responses to arginine in islets were similar to those in normal controls. GK rat is characterized by progressive loss of beta cells in the pancreatic islets with fibrosis. In GK rats, the percentage of GLUT2-positive per insulin-positive cells ranged from 85% at 12 wk, 48% at 24 wk, and 34% at 48 wk. During euglycemic clamp performed at submaximal hyperinsulinemia, suppression of liver glucose production was less effective in the GK rats, whereas their overall glucose utilization was similar to that of the control group.

### Proinsulin: Key to Diabetes Pathogenesis and Prevention in NOD Mice

LEONARD C. HARRISON, NATHAN R. MARTINEZ, HIDEO NIWA and RAYMOND J. STEPTOE

*The Walter and Eliza Hall Institute of Medical Research, Parkville 3050, Victoria, Australia*

Proinsulin is the only β-cell-specific autoantigen in type 1 diabetes. Several lines of evidence indicate that immunity to proinsulin drives β-cell destruction in NOD mice: 1) transgenic mice expressing proinsulin in antigen presenting cells are protected from insulitis and diabetes (diabetogenic T cells cannot be generated from the thymus and peripheral myeloid dendritic cells transfer protection), 2) proinsulin and a peptide that spans the B-C chain junction of proinsulin are targets of splenic T-cell reactivity in NOD mice at weaning, 3) T-cell clones isolated from the islets of NOD mice react to (pro)insulin epitopes, 4) administration of (pro)insulin protein or peptides by 'tolerogenic' modes or routes prevents diabetes development.

Our recent studies demonstrate that the proinsulin B-C chain peptide is presented by NOD dendritic cells and contains overlapping MHC class II (I-$A^{g7}$) and class I ($K^d$) epitopes. Mucosal (intranasal) administration of this peptide induces both regulatory CD4 T cells that prevent adoptive transfer of diabetes and CD8 cytotoxic T lymphocytes (CTL). Administration of a truncated peptide that only binds I-$A^{g7}$, or concomitant blockade of CD40-CD40L, averts CTL and markedly improves protection from diabetes. The mechanisms involved in generating the (normally cleaved) B-C epitope are unknown, but we have identified an uncleaved B-C chain of proinsulin II in pancreas and thymus formed by aberrant RNA splicing. In summary: CD4 and CD8 T-cell epitopes spanning the proinsulin B-C chain are key immune targets and therapeutic tools in NOD mice.

### Cause, Prevention and Cure of Autoimmune Diabetes in NOD Mice

JI-WON YOON

*Julia McFarlan Diabetes Research Centre, University of Calgary, Calgary, Alberta, Canada*

Type 1 diabetes results from the destruction of pancreatic β cells by β cell-specific autoimmune responses. Animal models of human type 1 diabetes such as the BB rat and NOD mouse have enhanced our understanding of pathogenic mechanisms of this disease. Cumulative evidence indicates that β cell autoantigens, macrophages, dendritic cells, B cells and T cells are involved in β cell-specific autoimmune responses. Macrophages play an essential role in the development and activation of β cell-cytotoxic T cells in NOD mice. Cytokines secreted by immunocytes including macrophages and T cells may regulate the direction of the immune response toward Th1 or Th2 T cells. Pancreatic β cells are destroyed by apoptosis through fas-fasL and TNF-TNF receptor interactions and by granzyme and perforin released from cytotoxic effector T cells. T cell-mediated autoimmune diabetes can be prevented by the control of finely tuned immune balance. Permanent remission of type 1 diabetes in NOD mice can be achieved by a novel insulin gene therapy. The gene therapy uses a recombinant adeno-associated virus that expresses a modified insulin, which possesses insulin activity without requiring enzymatic processing, under the control of the hepatocyte-specific L-type pyruvate kinase promoter, which regulates expression of the modified insulin analog in response to blood glucose levels. A single injection of this gene construct resulted in the long-term remission of autoimmune diabetes in NOD mice without any apparent side effects. Topics discussed will be: the role of β cell autoantigens, macrophages and T cells in the initiation and progression of pancreatic β cell destruction, the prevention of autoimmunity by immunoregulation, and the complete remission of autoimmune diabetes by insulin gene therapy.

### Molecular Genetics of Type 1 Diabetes in NOD Mice

HIROSHI IKEGAMI[a], TOMOMI FUMISAWA[a], SUSUMU MAKINO[b] and TOSHIO OGIHARA[b]

[a]*Department of Geriatric Medicine, Osaka University Graduate School of Medicine, Suita, Osaka,*
[b]*Shionogi Aburahi Laboratories, Shiga, Japan*

Type 1 diabetes is a multifactorial disease with both genetic and environmental factors contributing to the disease development. The genetic component is determined by multiple susceptibility genes. Among these, *Idd1* on chromosome 17 and *Idd3* on chromosome 3 confer strong susceptibility to type 1 diabetes in the NOD mouse. A and E genes in the class II MHC, and *Il2* gene encoding interleukin 2 are strong candidate genes for *Idd1* and *Idd3*, respectively. To prove that these genes are responsible for disease susceptibility, we used the ancestral haplotype congenic mapping strategy (Ikegami H., *et al.*, J Clin Invest, 1995), in which recombinant chromosomes containing the same candidate mutations as the NOD mouse, but different flanking markers from the NOD mouse, are introgressed onto NOD background genes to make NOD strains congenic for the recombinant chromosomes. Such recombinant chromosomes were successfully obtained in NOD-related strains derived from the same outbred colony as the NOD mouse: the CTS (Cataract Shionogi) and IIS (Inbred ICR Shionogi) strains for *Idd1*and *Idd3*, respectively. The NOD strain congenic for the recombinant *Idd1* interval from the CTS mouse developed type 1 diabetes, but the incidence was significantly lower and the age-at-onset was significantly later than those in NOD parental strains despite the presence of the *Idd1* candidate *A* and *E* genes identical to those in the NOD mouse. These data indicate that the A and E genes are not sufficient for MHC-linked susceptibility to type 1 diabetes and the second component of MHC-linked susceptibility gene (*Idd16*) is mapped to the region adjacent to, but distinct from class II A and E loci. In contrast, phenotypes of the NOD strain congenic for the recombinant *Idd3* interval from the IIS mouse were indistinguishable from the NOD parental strain, suggesting that *Il2* is responsible for *Idd3* effect. These data indicate the power and importance of congenic mapping in genetic dissection of multifactorial diseases.

### Beacon: A Novel Gene Involved in the Regulation of Energy Balance

G. R. COLLIER, J. McMILLAN, K. WINDMILL, K. WALDER, J. TREVASKIS,
S. JONES, A. DE SILVA, J. JOWETT, G. AUGERT, A. CIVITARESE and P. ZIMMET

*Metabolic Research Unit, Deakin University, Geelong, Victoria 3217, Australia*

The hypothalamus plays a major role in the control of energy balance via the coordination of several neuropeptides with their receptors. We utilized a unique polugenic animal model of obesity, *Psammomys obesus*, which exhibits a wide range of body weight, body fat content and metabolic changes when fed *ad libitum* a diet of normal laboratory chow. We performed differential display PCR on hypothalamic mRNA samples from lean and obese animals to identify novel genes involved in obesity. Here we describe a novel gene overexpressed in obese animals that encodes a small (73 amino acid) protein which we have called beacon. Beacon mRNA gene expression in the hypothalamus was measured using TaqMan PCR and was positively correlated with percentage body fat ($p < 0.01$) and body weight in *Psammomys obesus* ($p < 0.05$). Beacon was also expressed in all tissues examined including liver, pancreas, adipose tissue and muscle of *Psammomys obesus*, but expression levels were not related to body weight or body fat content in these tissues. Beacon gene sequencing in lean and obese *Psammomys obesus* revealed no differences in the coding regions. Amino acid sequences deduced from human mouse EST's had 100% identity with *Psammomys obesus* beacon, indicating that this protein is highly conserved between species. *In vivo* data suggest a role for beacon in the regulation of energy balance and body weight homeostasis that may be mediated, at least in part, through the NPY pathway. We propose that beacon may represent a new therapeutic target for the development of treatments for obesity.

### Fatty Acids and Muscle Insulin Resistance: New Insights from Animal Models

E. W. KRAEGEN, G. J. COONEY, J.-M. YE and S. M. FURLER

*Garvan Institute, Sydney, Australia*

This review considers evidence for, and putative mechanisms of lipid-induced muscle insulin resistance. We have used a range of animal models, including high fat feeding, and intravenous infusion of triglyceride/heparin or glucose which lead to accumulation of muscle lipid and development of insulin resistance. There are now quite plausible mechanistic links between muscle lipid accumulation and insulin resistance, which go beyond the classic Randle glucose-fatty acid cycle. Recent studies suggest that lipid-induced muscle insulin resistance is quite labile and easily reversed in other normal rodents. We postulate that muscle cytosolic accumulation of the active form of lipid, the long chain fatty acyl CoAs, is involved, leading to insulin resistance and impaired insulin signalling or impaired enzyme activity (*e.g.*, glycogen synthase or hexokinase) either directly or via chronic translocation/activation of mediators such as a protein kinase C. In rodents there are similarities in the pattern of muscle lipid accumulation/PKC translocation/altered insulin signalling/insulin resistance inducible by 3–5 h acute FFA elevation, 1–4 days intravenous glucose infusion or several weeks of high fat feeding. Actions of fatty acids to bind specific nuclear transcription factors provide another mechanism whereby different lipids could influence metabolism. The interactions described here are fundamental to understanding metabolic effects of PPAR activators which alter lipid metabolism and improve muscle insulin sensitivity in insulin resistant states.

### Nutritional Modification of Insulin Resistance in OLETF Rats

YUTAKA MORI

*Department of Int. Med., National Higashi-Utsunomiya Hospital, Tochigi, Japan*

We investigated the effect of long-term administration of highly purified eicopapentaenoic acid ethyl ester (EPA-E), an n-3 polyunsaturated fatty acid derived from fish oil, in comparison to the effects of lard, olive oil, safflower oil, or distilled water as the control on the development of insulin resistance in Otsuka Long-Evans Tokushima Fatty (OLETF) rats. After 17 or 18 weeks of treatment, the glucose infusion rate (GIR) in the euglycemic insulin-glucose clamp test showed a significant increase only in EPA-E treated rats compared with the control rats. The GIR in EPA-E treated animals was approximately three times greater than in the controls. Fatty acid analysis of phospholipids in skeletal muscles showed a significant increase of the C18:2, C20:5, and C22:5 components in EPA-E treated rats and, conversely, a significant decrease in C20:4. EPA-E treated rats showed a significant increase in GLUT4 mRNA in skeletal muscle when compared with control rats. Our results indicate that the beneficial effect of EPA-E on insulin resistance in OLETF rats is likely to depend on modification of the phospholipid components of the skeletal muscle membrane. In addition, after 12 months of treatment, the atherosclerotic changes of coronary artery were significantly improved pathohistologically in EPA-E-treated rats compared with those in control rats. These findings suggest that dietary fatty acids may play an important role in the development of insulin resistance and atherosclerosis in patients with type 2 diabetes.

### History of Derivation and Characteristics of KK Strain

SHIGEHISA TAKETOMI and HITOSHI IKEDA

*Pharmaceutical Research Division, Takeda Chemical Ind., Ltd., Osaka, Japan*

Kondo *et al.* (1957) selected out and established many mouse strains from Japanese native mice. Among these inbred mouse strains, Nakamura (1962) found that the KK mouse strain, which named "KK" for its habitat (Kasukabe in Saitama prefecture), is spontaneously diabetic.

Several investigators (Nakamura *et al.*, 1962 and 1967, Iwatsuka *et al.*, 1970, Dulin *et al.*, 1983) reported that the diabetic characteristics are moderate obesity (30–35 g body weight at the age of 5 months), sluggishness, polyphagia, polyuria, persistent glucosuria, glucose intolerance, moderate hyperglycemia, hyperlipidemia, insulin resistance of peripheral tissues, hyperinsulinemia, histological changes in the pancreas and renal glomerular changes, pointing out several similarities between the diabetic state in the KK mice and Type 2 diabetes associated with obesity.

A genetic study of KK mice indicated that diabetic traits were inherited by polygenes (Nakamura *et al.*, 1963). Because diabetes and obesity in KK mice is relatively moderate, Nishimura (1969), one of Kondo's coworkers transferred the yellow obese gene ($A^y$) into KK mice by the repeated crossing of yellow obese mice and KK mice. $A^y$ allele (dominant alleles at the mouse agouti locus) is associated phenotypically with yellow fur, hyperphagia and obesity. This congenic strain of KK mice has been named yellow KK or $KKA^y$ mice. The diabetic characteristics such as hyperglycemia, hyperinsulinemia and obesity of $KKA^y$ mice were observed at young ages (6–8 weeks).

Therefore, KK strain has a latent diabetic state before the development of hyperglycemia and glucosuria. At this stage, glucose intolerance and insulin resistance are already observed. KK mice become severely diabetic with aging and/or with increasing body weight induced by feeding high energy diets, by injection of goldthioglucose or introduction of the yellow obese gene $A^y$.

Recently, KK and $KKA^y$ mice are used for evaluation of antidiabetic compounds. Especially, thiazolidinediones (TZDs), a high affinity ligand for transcription factor PPAR $\gamma$ (peroxisome-proliferation activated receptor $\gamma$), were discovered in the vivo screening system using $KKA^y$ mice.

### GK Rats

KEN-ICHI SUZUKI and YOSHIO GOTO

*Department of Metabolism and Diabetes Mellitus, Tohoku Kosei-nenkin Hospital, Sendai, Miyagi, Japan*

Impaired insulin response, insulin resistance or both characterize type 2 diabetes mellitus. GK rat is a non-obese, mildly diabetic rat that was developed by inbreeding Wistar rats with selection of rats in each generation with the highest blood glucose levels during an oral glucose tolerance test. Total pancreatic insulin stores in GK rats were depleted by 60% in adult hyperglycemic GK rats compared to those of normal Wistar rats, and beta-cell mass in adult GK rats was associated with a noticeable alteration in the architecture of a subpopulation of islets: large islets displayed signs of disorganization of the mantle-core relationship due to prominent fibrosis, with clusters of beta cells widely separated by strands of connective tissue. The insulin secretion stimulated by glucose is markedly impaired in GK rats. In perifusion, glucose-stimulated insulin release was reduced by 90% for first phase and by 75% for second phase. The responses to arginine in islets were similar to those in normal controls. GK rat is characterized by progressive loss of beta cells in the pancreatic islets with fibrosis. In GK rats, the percentage of GLUT2-

positive per insulin-positive cells ranged from 85% at 12 wk, 48% at 24 wk, and 34% at 48 wk. During euglycemic clamp performed at submaximal hyperinsulinemia, suppression of liver glucose production was less effective in the GK rats, whereas their overall glucose utilization was similar to that of the control group. These data suggest a possible existence of insulin resistance in GK rats.

### The NOD Mouse, an Animal Model of Type 1 Diabetes: Establishment, Characteristics, and Pathogenesis

S. MAKINO[a], K. KUNIMOTO[a], M. HARADA[a], Y. MURAOKA[a], Y. KISHIMOTO[a], K. KATAGIRI[a], Y. TOCHINO[a], M. HATTORI[b] and H. IKEGAMI[c]

[a]*Shionogi Aburahi Laboratories, Shiga, Japan,* [b]*Joslin Diabetes Center, Boston, MA, USA,* [c]*Osaka University Medical School, Osaka, Japan*

In 1974, the discovery of a female mouse exhibiting polyuria, severe glycosuria, and rapid weight loss triggered the development of the non-obese diabetic (NOD) mouse. At that time, no suitable animal models for Type 1 diabetes had been available. Selective breeding using the offspring of the female mouse derived from outbred ICR mice was started, and the NOD mouse was established as an inbred animal model for Type 1 diabetes in Shionogi Aburahi Laboratories in 1980.

NOD mice spontaneously develop autoimmune Type 1 diabetes. The prominent characteristics are insulin deficiency, rapid weight loss after the onset of overt diabetes and eventual death if not treated with insulin. The clinical onset of overt diabetes is acute, but autoimmune process is chronic with infiltration of mononuclear cells into the pancreátic islets (insulitis) started long before the onset of overt diabetes. Infiltration of mononuclear cells is also observed in the submandibular, lacrimal and Harderians' glands.

Inheritance of Type 1 diabetes in NOD mice is multifactorial. The contribution of both genetic and environmental factors to disease susceptibility has been demonstrated. At present at least 18 susceptibility loci have been mapped to the mouse genome. Among these, the MHC-linked gene, especially those in Class II MHC region, confer the strongest susceptibility to the disease. Immunologically, T cells play an important role for the development of insulitis and overt diabetes.

The NOD mouse is etiologically an ideal model of organ-specific autoimmune diseases.

### The NSY Mouse: An Animal Model of Type 2 Diabetes with Polygenic Inheritance

HIROSHI IKEGAMI[a], HIRONORI UEDA[a] and MASAO SHIBATA[b]

[a]*Department of Geriatric Medicine, Osaka University Graduate School of Medicine, Osaka,* [b]*Department of Health, Aichi-Gakuin University, College of General Education, Aichi Japan*

The Nagoya-Shibata-Yasuda (NSY) mouse strain was established as an inbred animal model with spontaneous development of type 2 diabetes, by selective breeding for glucose intolerance from outbred Jcl:ICR mice. NSY mice closely mimic human type 2 diabetes in that the onset is age-dependent, the animals are not severely obese, and both insulin resistance and impaired insulin response to glucose contribute to disease development. Almost all male NSY mice develop type 2 diabetes by 48 weeks of age. Phenotypic characteristics of NSY mice related to type 2 diabetes include slightly higher body weight and intra-abdominal fat weight than those of control mice, hyperglycemia on fasting and after glucose challenge, fasting hyperinsulinemia, insulin resistance and impaired insulin secretion to glucose. These phenotypes were enhanced by environmental factors, such as sucrose supplementation and high fat diet, only in NSY, but not in control mice, suggesting gene-environmental interaction in NSY mice. Inheritance of type 2 diabetes in NSY mice is polygenic with at least three major loci (Nidd1n, Nidd2n and Nidd3n) on different chromosomes (Chr. 11, 14 and 6, respectively) contributing to disease susceptibility. When chromosome 11 from the NSY mouse was introgressed onto control C3H/He mice, the resulting consomic C3H.NSY-chr11

strain showed significantly higher blood glucose levels on fasting and after glucose challenge, significantly higher fasting plasma insulin level and lower insulin response to glucose than control C3H mice. In contrast, C3H.NSY-chr14 strain, in which chromosome 14 from the NSY mouse was introgressed onto control C3H mice, showed fasting hyperinsulinemia, but not hyperglycemia. Body weight and weight of epididymal fat pads in both consomic strains were not different from those in control C3H mice, suggesting that Nidd1n and Nidd2n affect diabetes-related phenotypes including insulin resistance independent of obesity. NSY mice is expected to be a useful model for studies on the etiology and genetics of late-onset type 2 diabetes with polygenic inheritance in humans.

### *References*

[1] Shibata, M. and Yasuda, B. (1980). New experimental congenital diabetes mice (N.S.Y. mice). *Tohoku J. Exp. Med.*, **130**, 139–142.
[2] Ueda, H., Ikegami, H. *et al.* (1995). The NSY mice: a new animal model of spontaneous NIDDM with moderate obesity. *Diabetologia*, **38**, 503–508.
[3] Ueda, H., Ikegami, H. *et al.* (1999). Genetic analysis of late-onset type 2 diabetes in a mouse model of human complex trait. *Diabetes*, **48**, 1168–1174.
[4] Ueda, H., Ikegami, H. *et al.* (2000). Age-dependent changes in phenotypes and candidate gene analysis in a polygenic animal model of type II diabetes mellitus; NSY mouse. *Diabetologia*, **43**, 932–938.
[5] Ueda, H., Ikegami, H. *et al.* (2000). Paternal-maternal effects on phenotypic characteristics in spontaneously diabetic Nagoya-Shibata-Yasuda mice. *Metabolism*, **49**, 651–656.

### Characteristics of the Spontaneous Occurring Diabetes (WBN/Kob) Rat

KAZUMASA NAKAMA and TOSHIO AKIMOTO

*Division of Laboratory Animal Sciences, Nippon Medical School*

WBN/Kob rats were derived from Wistar rat strain. The rats were brought to Japan by Dr. Kobori from the Bonn University in 1976, and named WBN/Kob (Wistar Bonn/Kobori). In 1980, Tobe and coworkers, in an attempt to clarify biological characteristics of the rats, carried out a long-term observation of these rats and found that at the age of about 9 months, some male rats manifested polyuria and glycosuria, and developed a marked glucose intolerance. Thereafter, Nakama *et al.* and Tsuchitani *et al.* carried out observation to clarify further details of the pathophysiological aspect of the disease independently, and reported, at the same time in 1985, that the WBN/Kob male rats manifest the diabetes without obesity caused by a peculiar pancreatic disease developed in the rats spontaneously. Since then, the features of this disease were revealed by a number of reports, and it has been recognized that WBN/Kob male rats could be used as a model for study on the pathogenesis of non-obese type 2 diabetes and pancreatitis.

Diabetes develops in some male rats at about 36 weeks of age, and in all at the age of about 68 weeks, spontaneously. The male rats manifest an overt diabetes with a moderate degree of the disease condition (non-fasted blood glucose level: 300–450 mg/dl) after a relatively long-term duration of the glucose intolerance stage, and are able to survive for a long time without any insulin medication. Histopathologic examination of the pancreas revealed severe changes in male rats. These changes start as early as 8 weeks of age as interstitial edema of the exocrine parenchyma with infiltration of inflammatory cells, erythrocytes, fibroblasts, and deposition of hemosiderin. Between 12 and 24 weeks of age marked fibrosis is seen around the pancreatic ducts and blood vessels. With advancing age the fibrous tissue gradually invades extensive areas of the pancreas where also the islets become involved in fibrotic degeneration. At 68 weeks of age and later, an obvious decrease in islet number and size has been observed even in unaffected organs. Females do not develop this pathology. The complication so the diabetes have been noted in kidney, eye and peripheral nerve. The renal lesion has been observed as the exudative lesion of the glomeruli at 24 weeks of age and later. Thickening of the basement membrane, increase of the mesangial matrix and fibrin-cap lesions have been noted in the glomeruli. Armanni-Ebstein degeneration is occasionally found in the tubules. Frequent bilateral cataracts begin to appear at about 60 weeks of age. The lesions of peripheral nerve have been confirmed as nerve fiber detachment and myelin sheath swelling of the sciatic nerve.

The diabetes of WBN/Kob male rat is known to develop hereditarily. Recently, Nishimura *et al.* performed genetic linkage analysis of the pancreatitis and reported that the gene locus linkaged to pancreatitis were successfully mapped on rat Chr 7 and Chr X.

### The TSOD Mouse: A New Animal Model of Spontaneous NIDDM with Obesity

WATARU SUZUKI, SEIICHI IIZUKA, MASAHIRO TABUCHI,
TOSHIHIKO YANAGISAWA and MASAYUKI KIMURA

*Research & Drug Division, Tsumura & Co.*

The animal model that duplicates every feature of the diabetes mellitus in man is very useful for elucidation of the mechanisms of the disease. The TSOD (Tsumura, Suzuki, Obese Diabetes) mouse was established a spontaneous model of NIDDM with moderate obesity by selective breeding for urinary glucose and body weight.[1]

We found 6 obese male mice with urinary glucose in our stock (ddY strain) derived from Doken (Ibaraki) in 1984. Their offspring were used for brother-sister mating. In the inbreeding, the animals with the body weight at 8 weeks of age among litter-mates were selected for breeding and the males with intensive urinary glucose (at 8 to 40 weeks of age) were preferentially used.

By the selective breeding of obese male mice, we established two inbred strains in 1992: diabetic strain with the obesity and urinary glucose (TSOD) and the other without them (TSNO: Tsumura, Suzuki, Non Obesity). The male TSOD mice constantly showed signs of obesity and urinary glucose with increases in food and water intake, and the obesity resulted from increases in the intra-abdominal fat. The male TSOD mice because massively obese with weight ranging from 60 g to 70 g, which developed hyperglycemia and hyperinsulinemia, and impaired glucose tolerance with age. The levels of blood glucose and insulin were still high to the ages past the growth peak.

Histopathologically, severe hypertrophy of the pancreatic islets due to proliferation of B cells was observed without insulitis in almost all of the adult males. The hypertrophy of pancreatic islets was progressive with age although islets atrophy and fibrosis were not observed in aged mice. In the other organs (kidney, eye and peripheral nerve), aged TSOD males had some histopathological characteristic changes that might be due to persistent hyperglycemia and/or hyperinsulinemia.

Recently, through a whole genome scan of quantitative trait loci (QTLs) affecting body weight, blood glucose, and insulin levels of the TSOD mice, three major loci meeting the rigorous criteria for linkage were identified.[2] The major genetic determinant of blood glucose levels was identified on chromosome 11. Two independent QTLs involved in controlling body weight were found on chromosome 1 and 2. The QTL on chromosome 2 also affected insulin levels. These evidences suggest that the diabetic feature of the TSOD mouse is polygenic.

Among several animal models for NIDDM, the TSOD mouse shows progressive symptoms of diabetes with similarity to the NIDDM in man.

### References

[1] Suzuki, W., Iizuka, S., Tabuchi, M., Yanagisawa, T., Funo, S., Kimura, M., Sato, T., Endo, T. and Kawamura, H. (1999). A new mouse model of spontaneous diabetes derived from ddY strain. *Exp. Anim.*, **48(3)**, 181–189.

[2] Hirayama, I., Yi, Z., Izumi, S., Arai, I., Suzuki, W., Nagamachi, Y., Kuwano, H., Takeuchi, T., and Izumi, T. (1999). Analysis of obese diabetes in the TSOD mouse. *Diabetes*, **48**, 1183–1191.

### The OLETF Rat As a Model for Human Type II Diabetes

TSUKASA HIRASHIMA and KAZUYA KAWANO

*Department of Research Management, Otsuka Pharmaceutical Co., Ltd., Tokushima, Japan*

A spontaneous polyphagia, polydipsia, polyuria and mild obesity was discovered in 1984 in an outbred colony of Long Evans (LE) rats, that had been purchased from Charles River Canada in 1982. A strain of rats was developed by selective breeding from this rat is now referred to as the Otsuka Long-Evans Tokushima Fatty (OLETF) rat. From the same LE colony, a line developing type I diabetes has been isolated (LETL) and a control line, LETO, has also been established.

In this panel session, we will present the characteristic features of OLETF rats and briefly summarized as follows: 1) After 24 wk of age, elevation of plasma glucose during the oral glucose tolerance test (OGTT), became marked, indicating diabetes mellitus. The cumulative incidences of diabetes and impaired glucose tolerance (IGT) were 87.8% (341 of 388) and 7.2% (28 of 388), respectively, in male OLETF rats after the 20th generation. Glucoseuria was observed approximately 30% of male OLETF rats after 40 wk of age. Female OLETF rats displayed diabetes in very few individuals at 30 wk of age, though approximately 30% of females showed diabetes when 60 wk old. 2) Plasma insulin level during the OGTT did not differ between the strains at 10 weeks of age. However, plasma insulin was markedly higher in the OLETF rat at 30 and 50 wk of age. In contrast, plasma insulin in 70-week-old OLETF rat with severe diabetes was even lower than the LETO rats. 3) The average daily food intake for an OLETF rat is approximately 30% more than LETO rats from 6 wk of age. 4) Mild obesity accompanied with visceral fat accumulation was observed. 5) Plasma triglyceride level in OLETF rats was significantly higher than LETO rats from 6 wk of age and the difference progressively increased with age. Then hypertriglyceridemia resulted in significant TG stores in the islets, liver, and muscle, plays an important role in the development of diabetes in OLETF rats. 6) Insulin resistance in skeletal muscle and impaired insulin secretion were appeared from 12 wk of age.

Since the clinical features of the disease state in OLETF rats resemble those of human type II diabetes, OLETF rats should be a useful model for analysis of the pathogenesis and complications of type II diabetes and for studies on the development of new diabetic medicine.

### Characterization of a New Spontaneously Diabetic Non-Obese Torii Rat Strain

MASAMI SHINOHARA[a], TAKU MASUYAMA[a], TOSHIYUKU SHODA[a], KAJURO KOMEDA[b], AKIHIRO KAKEHASHI[c], MASATOSHI KUROKI[c] and YASUNORI KANAZAWA[c]

[a]*Torii Pharmaceutical Co., Ltd.*, [b]*Division of Laboratory Animal Science, Animal Research Center, Tokyo Medical University,* [c]*Omiya Medical Center, Jichi Medical School*

In 1988, five male rats with polyuria, glucosuria, polyphagia, and polydipsia were identified among 305 rats from an outbred colony of the Crj:CD(SD) strain of Sprague-Dawley rats, which were kept in the Research Laboratories of Torii Pharmaceutical Co., Ltd. After the 20th generation of sister-brother mating, the diabetic strain was established in 1997, and named the SDT (Spontaneously Diabetic Torii) rat. The time of onset of glucosuria was different between male and female SDT rats, glucosuria appeared at approximately 20 weeks of age in male rats and at approximately 45 weeks of age in female rats. A cumulative incidence of diabetes of 100% was noted by 40 weeks of age in male rats, while it was only 33.3% even by 65 weeks of age in female rats. The clinical characteristics of the male SDT rats were as follows: (1) impaired glucose tolerance (from 16 weeks of age); (2) hyperglycemia and hypoinsulinemia (from 25 weeks of age); (3) long-term survival without insulin treatment; (4) no obesity and (5) hypertriglyceridemia (by 35 weeks of age). The histopathological characteristics of the male rats were as follows: (1) hemorrhaging in and around the pancreatic islets (by 10 weeks of age); (2) fibrosis of the pancreatic islets (by 25 weeks of age); (3) Armanni-Ebstein lesion in the epithelial cells of the uriniferous tubules (by 35 weeks of age); (4) cataract (by 40 weeks of age); (5) tractional retinal detachment with fibrous proliferation (by 70 weeks of age) and (6) massive hemorrhaging in the anterior chamber (by 77 weeks of age). On the other hand, in the females, fibrosis around the pancreatic islets was observed at 40 weeks of age. These results suggest that the clinical and histopathological characteristics of SDT rat resemble those of human Type 2 diabetes with insulin hyposecretion. In conclusion, SDT rat is considered to be a potentially useful model for studies of diabetic complications encountered in humans.

### *Reference*

[1] Shinohara, M., Masuyama, T., Shoda, T., Takahashi, T., Katsuda, Y., Komeda, K., Kuroki, M., Kakehashi, A., and Kanazawa, Y. (2000). A new spontaneously diabetic non-obese Torii rat strain with severe ocular complications. *Int. Jnl. Experimental Diab. Res.*, **1**, 89–100.

### The KDP Rat: An Animal Model of Human Type 1 Diabetes

K. KOMEDA[a], N. YOKOI[b] and Y. KANAZAWA[c]

[a]*Tokyo Medical University, Tokyo,* [b]*Chiba University, Chiba,* [c]*Jichi Medical School, Saitama, Japan*

In 1991, the Long-Evans Tokushima Lean (LETL) rat was established as an animal model for human type 1 diabetes by selective inbreeding. LETL rats are characterized by the rapid onset of diabetes, absence of gender difference in the incidence of diabetes, autoimmune destruction of pancreatic β-cells, and absence of significant T lymphopenia. Although the LETL rats have been established as an inbred strain, the incidence of diabetes is only <20%. In 1998, we established two substrains, the Komeda diabetes-prone (KDP) and the Komeda non-diabetic (KND), from the original inbred LETL rats. The features of KDP rats are, the high incidence of diabetes (~70%), absence of lymphopenia and 100% development of mild to severe insulitis at 120–220 days of age. In contrast, the KND rat is characterized by the complete absence of diabetes incidence. Insulitis was found in KDP rat at 40 days of age. The first change observed in the pancreas was overexpression of the class I antigen in the vascular endothelium, exocrine cells and β-cells of the prediabetic KDP rat. Next, ED1+, CD4+ and CD5+ cells simultaneously infiltrated the islet of the KDP rat. A fewer number of CD8+ and CD45RA+ cells were also present and their numbers increased with severity of insulitis in the KDP rat. These infiltrating cells showed strong expression of class I and II antigens and intercellular adhesion molecule 1 (ICAM-1). However, the class II antigen was not observed in the β-cells. We have mapped a major diabetes locus, termed *Iddm/kdp1*, on rat chromosome (Chr) 11 using three backcross panels. Homozygosity for the KDP allele at this locus is shown to be essential for the development of moderate to severe insulitis and the onset of diabetes. Comparative mapping suggests that the homologues of *Iddm/kdp1* are located on human Chr 3 and mouse Chr 16. We are currently analyzing the genome of the *Iddm/kdp1* region in the KDP rat. The KDP rat should serve as an effective model for research on the pathogenesis and for genetic analysis of type 1 diabetes in humans.

### *References*

[1] Kawano, K., Hirashima, T., Mori, S., Saitoh, Y., Kurosumi, M. and Natori, T. (1991). New inbred strain of Long-Evans Tokushima Lean rats with IDDM without lymphopenia. *Diabetes*, **40**, 1375–1381.
[2] Yokoi, N., Kanazawa, M., Kitada, K., Tanaka, A., Kanazawa, Y., Suda, S., Ito, H., Serikawa, T. and Komeda, K. (1997). A non-MHC locus essential for autoimmune type I diabetes in the Komeda diabetes-prone rat. *J. Clin. Invest.*, **100**, 2015–2021.
[3] Komeda, K., Noda, M., Terao, K., Kuzuya, N., Kanazawa, M. and Kanazawa, Y. (1998). Establishment of two substrains, diabetes-prone and non-diabetic, from Long-Evans Tokushima Lean (LETL) rats. *Endocrine J.*, **45**, 737–744.

### ALS and ALR Mice Are Useful Diabetic Models to Reveal Correlations between Obesity and Diabetes

T. YAMASHITA and K. SATO

*Faculty of Agriculture, Okayama University, Okayama, Japan*

Both ALS; alloxan-induced diabetes-susceptible, strain and ALR; alloxan-induced diabetes-resistant, strain were derived from Crj: CD-1 (ICR) mice and developed as mouse models of alloxan-induced diabetes by Dr. Takayoshi Ino *et al.*[1–3] ALS and ALR strains were established as inbred strains by a two-way selection toward the high- and low-incidences of diabetes with alloxan administration (45 mg/kg in males, 47 mg/kg in females) in every generation. These strains respectively indicated distinct changes in the incidences such as diabetes and the blood glucose levels with generation. In the ALS strain, the incidence reached 98.9%, and the blood glucose level averaged 423 ± 11 mg/dl in the 13th generation. In the ALR strain, the incidence reached 0%, and the blood glucose levels showed 128 ± 4 mg/dl in the 7th generation. These strains did not show a spontaneous onset of diabetes. However, when an obesity gene ($A^y$) was introduced to these strains, diabetic conditions occurred spontaneously in these animals. Then ALS-$A^y$ and ALR-$A^y$ strains were developed as congenic strains produced by introducing the $A^y$

gene. In comparison with ALS mice, male ALS-$A^y$ mice had no obesity, but showed very severe diabetic conditions with very low glucose tolerance and insulin secretion. On the other hand, female ALS-$A^y$mice were obese and showed severe diabetic conditions with no marked decrease in glucose tolerance and high insulin secretion. Both male and female, ALR-$A^y$ mice were obese and showed diabetic conditions, though the severity of the diabetic conditions tended to be lower than those of ALS-$A^y$ mice. Furthermore, we induced ALS and ALR mice to be obese by neonatal treatment of monosodium-L-aspartate (MSA). As a result, all of MSA-treated ALS and ALR mice were obese. MSA-treated male ALS mice showed very severe diabetic conditions with very low insulin secretion, and began to become lean after 8 weeks of age with disappearance of pancreatic islets. MSA-treated female ALS mice showed severe diabetic conditions with high insulin secretion and hyperplasia of pancreatic islets. In contrast, both male and female, MSA-treated ALR mice did not show diabetic conditions. The characteristic features of diabetic conditions which appeared in these mice suggest that these strains serve as useful models to reveal correlations between obesity and diabetes.

### *References*

[1] Sekiguchi, F., Ishibashi, K., Katoh, H, Kawamoto, Y. and Ino, T. (1990). Genetic profile of alloxan-induced diabetes-susceptible mice (ALS) and -resistant mice (ALR). *Exp. Anim.*, **39**, 269–272.
[2] Ino, T., Kawamoto, Y., Sato, K., Nishikawa, K., Yamada, A., Ishibashi, K. and Sekiguchi, F. (1991). Selection of mouse strains showing high and low incidences of alloxan-induced diabetes. *Exp. Anim.*, **40(1)**, 61–67.
[3] Sekiguchi, F., Ishibashi, K., Kawamoto, Y. and Ino, T. (1991). Diabetic peculiarity of the ALS-$A^y$ and ALR-$A^y$ strains. *Exp. Anim.*, **40**, 323–329.

### Characterization of Akita Mouse

AKIO KOIZUMI

*Department of Health and Environmental Health Sciences, Kyoto University School of Public Health, Kyoto 606-8501, Japan*

The Akita mouse, which has a mutation (Cys 96 Tyr) in the insulin 2 gene ($Ins2^{Akita}$+/–), is a model for diabetes. The male Akita mouse ($Ins2^{Akita}$+/–) has profound diabetes from very early age, onset by 10 weeks of age, while females ($Ins2^{Akita}$+/–) remain only mildly diabetic throughout life. Although mutation of the Ins2 gene has a causative role in diabetes molecular pathogenesis remains unknown. In this abstract characteristics of the diabetes in Akita mouse are presented together with unsolved problems, the elucidation of which may give an insight into the quality control of protein processing not only in _-cell but also many other cells.

**Characterization of Akita mouse**

1. Profile of the animal model: Mutation of *Ins2* (C96T) causes diabetes. Mice were maintained in a heterozygous state on either C57BL/6 or C3H/He background strains.
2. Characterization:
   Incidence of diabetes: There is a profound gender difference: diabetes becomes evident from 6 weeks of age in males but remains mild in females. Females, however, have impaired glucose tolerance by IPGTT.
   Physiological characteristics: Impaired insulin production.
   Morphological changes: β_-cell hypoplasia in the pancreas.
3. Complication: Renal complications with IgA nephropathy.
4. Genetics: Autosomal dominant.

**Comments:** Gene dosage effect predicts that a mutation of one of the Ins2 alleles simply decreases the amount of insulin production by 25%. This contradicts the evidence in the Akita mouse in which such a mutation causes diabetes. One explanation of the diabetic phenotype in the Akita mouse is that the missense mutation of cysteine (A7) to tyrosine destroys the A7-B7 disulfide bridge and thereby results in impaired folding of insulin. It is, however, entirely unknown how misfolded insulin impairs _-cells function and inhibits _-cell proliferation. It is also entirely unknown why underlying pathological processes are mitigated in females. The Akita mouse is not only a model for diabetes but also is a model for the processing of abnormal proteins in β-cells.

### References

[1] Yoshioka, M., Kayo, T., Ikeda, T. and Koizumi, A. (1997). A novel locus, Mody 4, distal to D7Mit189 on chromosome 7 determines early-onset NIDDM in nonobese C57BL/6 (Akita) mutant mice. *Diabetes*, **46**, 887–894.
[2] Kayo, T. and Koizumi, A. (1998). Mapping of murine diabetogenic gene *Mody* on chromosome 7 at D7Mit258 and its involvement in pancreatic islet and β cell development during the perinatal period. *J. Clin. Invest.*, **101**, 2112–2118.
[3] Wang, J., Takeuchi, T., Tanaka, S., Kubo S.-K., Kayo, T., Lu, D., Takata, K., Koizumi, A. and Izumi, T. (1999). A mutation in the insulin 2 gene induces diabetes with severe pancreatic β-cell dysfunction in the Mody mouse. *J. Clin. Invest.*, **103**, 27–37.
[4] Fujita, H., Haseyama, T., Kayo, T., Nozaki, J., Wada, Y., Ito, S. and Koizumi, A. (In press). An increased expression of glutahione S-transferases in the renal proximal tubuli in the early stage of diabetes: a study in a mouse model of type 2 diabetes Akita mouse. *Exp. Nephrol.*

### Establishment and Characteristics of the Wistar Fatty Rat

HIROYUKI ODAKA and HITOSHI IKEDA

*Pharmaceutical Research Division, Takeda Chemical Ind., Ltd., Osaka, Japan*

It is well-known that the phenotypes often depend on the genetic background. Our previous findings in three mouse strains clearly indicated a close relationship between genetic and environmental factors n the diabetogenic action of obesity. Dietary obesity induced hyperglycemia only in KK mice, while goldthioglucose-induced obesity was associated with hyperglycemia in KK and ICR mice but not in C57BL/6 mice. The difference observed among these mouse strains could be accounted for by genetic factors manifested as the degree of glucose intolerance: KK > ICR > C57BL/6. Therefore, we thought glucose intolerance and insulin resistance were good makers for predisposition to diabetes. The Zucker fatty rats (*fa/fa*, ZF) are obese but normoglycemic. Based on the above-mentioned concept, the *fa*-gene was transferred from the *fa*/+ Zucker rat (13M strain) to the Wistar Kyoto (WKY) rat strain that has less sensitivity to insulin and less tolerance to glucose. At the $5^{th}$ generation of repeated backcrossing of *fa*/+ WKY-Zucker hybrids to WKY, male obese hybrid rats were found to be hyperglycemic. This finding clearly demonstrated our concept. The survey, performed at the $10^{th}$ generation of backcrossing, showed that the Wistar fatty rat (*fa/fa*, WF), a congenic strain of WKY, developed obesity and obesity-related features, such as hyperinsulinemia and hyperlipemia, in the same manner as ZF rats. Male, but not females, showed hyperglycemia, glucosuria, and polyuria as early as 8 weeks of age. Tolerance and insulin response to oral glucose decreased with advancing age in males. Male WF rats also showed the hepatic and peripheral insulin resistance. Therefore, WF rats were confirmed to be a good model for human type 2 diabetes mellitus with insulin resistance and used to evaluate efficacy and the mechanism of insulin sensitizer, pioglitazone.

### Overexpression of Dominant Negative HNF-1α in β-Cells Affects the Morphogenesis of Islets and the Proliferation of β-Cells

TAKAO NAMMO, KAZUYA YAMAGATA, MAKOTO MORIWAKI, ARISA IHARA, MING LI, QIN YANG, RIKAKO UENAKA, KOHEI OKITA, HIROMI IWAHASHI, QIAN ZHU, YANG CAO, AKIHIS IMAGAWA, JUN-ICHIRO MIYAGAWA and YUJI MATSUZAWA

*Department of Internal Medicine and Molecular Science, Graduate School of Medicine, B5, Osaka University, Suita, Osaka Japan*

Mutations in the gene encoding hepatocyte nuclear factor (HNF)-1α are the cause of the type 3 form of MODY (MODY3). To investigate the molecular mechanisms of MODY3, we generated transgenic (Tg) mice overexpressing dominant negative form of HNF-1α (P291fsinsC-HNF-1α), which is the most common mutation in HNF-1α, in β-cells under the control of rat insulin promoter. Tg mice developed diabetes around 4 weeks of age with impaired insulin secretion. Histological examinations revealed abnormal islet architecture and reduced β-cell number before the onset of diabetes. β-cells were found scattering among non β-cells in the Tg islets. Proliferation rate of β-cells measured by BrdU incorporation in the Tg mice was reduced by 15% compared to that in the control mice. Although these data suggest that HNF-1α is important for normal development of β-cells, expression of PDX-1 and Pax6 in Tg β-cells was not affected.

### The Role of Sialidase Gene on Insulin Resistance: Sialidase Transgenic Mice and Human Sialidase Gene Polymorphisms

Y. HINOKIO[a], S. SUZUKI[a], M. HIRAI[a], C. SUZUKI[a], J. SATOH[a], A. SASAKI[b], K. YAMAGUCHI[b] and T. MIYAGI[b]

[a]*Tohoku University School of Medicine,* [b]*Sendai and Miyagi Prefectural Cancer Center, Natori, Japan*

It has been previously reported that gangliosides and sialic acids play an important role in both type 1 and type 2 diabetes, and hypertension. Sialidase is a key enzyme removing sialic acids from gangliosides. We generated transgenic mice (TG) expressing plasma membrane-associated sialidase specific for gangliosides. After an intraperitoneal glucose tolerance test, they had hyperglycemia, and moreover, plasma insulin levels were increased 30-fold in TG compared with control mice. Insulin tolerance test showed insulin resistance in TG. Insulin-stimulated phosphorylations of insulin receptor were decreased, but glycogen synthase activity in skeletal muscle of TG were much higher than in those of control. Hematoxylin and eosin strain showed hyperplasia of pancreatic islets, and immunohistochemistry showed diffused stain with anti-insulin antibody. These results provide evidence that overexpression of plasma membrane-associated sialidase can lead to insulin resistance. To examine the association between human sialidase gene and diabetes, we screened polymorphisms and mutations in the gene in Japanese diabetic patients. We determined SNP (T/C) in exon3. The allele frequencies of the SNP were different between diabetic group and control group (diabetes: C 0.596, T 0.404; control: C 0.751, T 0.249) ($p < 0.001$). And moreover, we determined mutation (A to G) converting lysine to arginine in exon3. This mutation was not found both in the all other diabetics and controls. From these results, it may be concluded that sialidase gene may play an important role in diabetes mellitus and insulin resistance.

### Increased Expression of the Sterol Regulatory Element-Binding Protein (SREBP)-1 Gene in Insulin Receptor Substrate-$2^{-/-}$ Mouse Liver

RYO SUZUKI[a], KAZUYUKI TOBE[a], MASASHI AOYAMA[a], TOSHIMASA YAMAUCHI[a], JUNJI KAMON[a], NAOTO KUBOTA[a], YASUO TERAUCHI[a], JUNJI MATSUI[a], YASUO AKANUMA[b], KAJURO KOMEDA[c], SATOSHI KIMURA[a], JUN TAKAKA[d], MANABU ABE[d], JUN OHSUMI[d], RYOZO NAGAI[a] and TAKASHI KADOWAKI[a]

[a]*The Department of Internal Medicine, Graduate School of Medicine, University of Tokyo, Japan,* [b]*Institute for Diabetes Care and Research, Asahi Life Foundation, Tokyo, Japan,* [c]*Animal Research Center, Tokyo Medical University, Tokyo, Japan,* [d]*Biomedical Research Laboratories, Sankyo Co. Ltd., Tokyo, Japan*

We previously demonstrated that insulin receptor substrate (IRS)-$2^{-/-}$ mice develop diabetes due to insulin resistance in the liver and failure to undergo β-cell hyperplasia. To understand the role of the liver in the development of diabetes in IRS-$2^{-/-}$ mice, we performed a global gene expression study by murine 11K oligonucleotide microarray (Affymetrix) using liver samples. Quite unexpectedly, remarkable induction of the sterol regulatory element-binding protein (SREBP)-1 gene, a downstream target of insulin, was detected in 16-week-old IRS-$2^{-/-}$ mouse liver, where insulin-mediated intracellular signaling events, including PI3-kinase activation, were substantially attenuated. Expression of SREBP -1 downstream genes, such as the spot 14, ATP citrate-lyase, fatty acid synthase, and malic enzyme genes, was also increased. RNase protection assay revealed that expression of SREBP-1c, but not SREBP-1a, was increased. All these findings were confirmed by Northern blot analysis. Liver triglyceride content was significantly increased in IRS-$2^{-/-}$ mice, assuring the physiological importance of SREBP-1 gene induction. SREBP-1 gene up-regulation was detectable even in 6-week-old euglycemic IRS-$2^{-/-}$ mouse liver. These young mice showed increased adiposity associated with hyperleptinemia. In addition, low-dose leptin administration, enough to reduce food intake and body weight in wild-type mice, failed to do so in IRS-$2^{-/-}$ mice, suggesting that IRS-$2^{-/-}$ mice had leptin resistance. Interestingly, high-dose leptin administration reduced SREBP-1 expression in IRS-$2^{-/-}$ mouse liver. Thus, IRS-2 gene disruption results in leptin resistance, causing a SREBP-1 gene induction, obesity, fatty liver, and diabetes.

### IRS-2 Deficient Mice Showed a Marked Exacerbation of Diabetes under a High-Fat Diet

KAZUYUKI TOBE[a], RYO SUZUKI[a], ASASHI AOYAMA[a], NAOTO KUBOTA[a], YASUO TERAUCHI[a], TOSHIMASA YAMAUCHI[a], JUNJI KAMON[a], KAJURO KOMEDA[b] and TAKASHI KADOWAKI[a]

*[a]The Department of Internal Medicine, Graduate School of Medicine, University of Tokyo,*
*[b]Animal Research Center, Tokyo Medical University*

We previously demonstrated that IRS-2(–/–) mice developed diabetes due to insulin resistance in liver and lack of compensatory β-cell hyperplasia. To investigate interactions between genetic and environmental factors in the development of type 2 diabetes, we studied the effect on high-fat (HF) diet on glucose metabolism in IRS-2(–/–) mice. 6 w/o male IRS-2(–/–) and wild-type mice were fed on an HF diet or a regular chow (RC) diet for 4 weeks. Mice under an HF diet showed more weight gain than mice under an RC diet. An HF diet-induced increase in adipose tissue mass in IRS-2(–/–) mice was almost comparable to that in wild-type mice. However, oral glucose tolerance test revealed that IRS-2(–/–) mice under an HF diet showed a marked exacerbation of glucose tolerance (blood glucose level at 0 min: IRS-2–/–HF 203 ± 5, IRS-2–/–RC 106 ± 9, WT-HF 98 ± 7, WT-RC 74 ± 5 mg/dl, 120 min: IRS-2–/–HF 481 ± 27, IRS-2–/–RC 174 ± 20, WT-HF 129 ± 6, WT-RC 94 ± 4 mg/dl). Insulin tolerance test showed that the glucose lowering effect of insulin was markedly aggravated under an HF diet in IRS-2(–/–) mice while HF diet-induced insulin resistance in wild-type mice was modest. To investigate the mechanism of HF diet-induced insulin resistance observed in IRS-2(–/–) mice, the expression of enzymes in the gluconeogenic pathway in liver was examined. As IRS-2(–/–) mice showed defects in insulin signaling in liver, suppression of PEPCK and G6Pase gene expression in the fed state was impaired in IRS-2(–/–) mice. Under an HF diet, PEPCK gene expression in the fasted state was significantly increased in IRS-2(–/–) mice compared with that in wild-type mice. We concluded that a high-fat diet contributes largely to the development of insulin resistance and diabetes when genetic factors of type 2 diabetes exist.

### Impact of Genetic Background and Ablation of IRS-3 on IRS-2 Knockout Mice

YASUO TERAUCHI, NAOTO KUBOTA, JUNJI MATSUI, KAJURO KOMEDA, KAZUYUKI TOBE, GUSTAV E. LIENHARD and TAKASHI KADOWAKI

*Tokyo, Japan; Dartmouth, NH, USA*

Although we and another group have generated IRS-2-knockout mice (IRS-$2^{-/-}$), a significant difference in the phenotypes of IRS-$2^{-/-}$ between the two groups was seen in the severity of diabetes. It has been reported that while IRS-1 and IRS-3-knockout mice (IRS-$3^{-/-}$) showed normal fasting plasma glucose (FPG) level, IRS-1/IRS-3 double knockout mice exhibited marked hyperglycemia. To assess the effect of genetic background and also ablation of IRS-3 on IRS-$2^{-/-}$, we generated IRS-2/IRS-3 double knockout mice (IRS-$2^{-/-}$IRS-$3^{-/-}$) by crossing IRS-$3^{-/-}$ (129/Sv × C57B1/6 background) with our IRS-$2^{-/-}$ (CBA × C57B1/6 background). Intercrosses of IRS-$2^{+/-}$IRS-$3^{+/-}$ yielded nine genotypes of mice. Out of 365 male offspring, 18 IRS-$2^{-/-}$IRS-$3^{-/-}$ males were viable. At 6 weeks of age, there were no differences in FPG levels among the nine genotypes of mice although IRS-$2^{-/-}$ showed elevated insulin levels. At 10 to 20 weeks of age, about 30% of IRS-$2^{-/-}$ developed overt diabetes. Thus, FPG levels were elevated to more than 350 mg/dl. It should be noted that IRS-$2^{-/-}$ on the CBA × C57B1/6 strain had never developed such severe type of diabetes. When mice with overt diabetes were excluded from the analysis of glucose and insulin tolerance test, IRS-$2^{-/-}$IRS-$3^{-/-}$ showed a similar degree of glucose intolerance/insulin resistance to IRS-$2^{-/-}$. The amount of pancreatic beta-cells in both IRS-$2^{-/-}$ and IRS-$2^{-/-}$IRS-$3^{-/-}$ was moderately reduced in spite of insulin resistance. Insulin-positive beta-cells were dramatically decreased to almost zero in IRS-$2^{-/-}$ with overt diabetes. Taken together, IRS-3 does not play a compensatory role for the loss of IRS-2 in maintaining glucose homeostasis. The severity of diabetes in IRS-$2^{-/-}$ was dependent upon genetic background, suggesting the existence of modifier gene(s) for diabetes in the 129/Sv mouse genetic strain.

### Increased Insulin Sensitivity Despite Lipodystrophy in Heterozygous CBP Deficient Mice

TOSHIMASA YAMAUCHI, JUNJI KAMON, HIRONORI WAKI, KAJURO KOMEDA, ATSUKO TSUCHIDA, TASUO AKANUMA, RYOZO NAGAI, SATOSHI KIMURA, YICHI OIKE, KENICHI YAMAMURA and TAKASHI KADOWAKI

*Department of Internal Medicine, Graduate School of Medicine, University of Tokyo, Tokyo 113-8655, Japan*

CBP (cAMP response element binding protein (CREB) binding protein) functions as a coactivator towards various transcriptional factors. Unexpectedly, heterozygous CBP deficient ($CBP^{+/-}$) mice showed markedly reduced weight of white adipose tissue (WAT), but not that of other tissues. Despite this lipodystrophy, $CBP^{+/-}$ mice showed increased insulin sensitivity and glucose tolerance, and were completely protected from high-fat diet-induced body weight gain. In $CBP^{+/-}$ mice, increased leptin sensitivity and increased serum adiponectin levels were observed, and these increased effects of insulin sensitizing hormones secreted from WAT may explain the phenotypes of $CBP^{+/-}$ mice at least in part. This study demonstrates that CBP may play a major role in the physiological regulation of energy storage, and that WAT could exert anti-obesity and anti-diabetic effects via endocrine rather than metabolic mechanisms.

### Treatment of NIDDM in the GK Rat Model by Expansion of the β-Cell Mass during the Prediabetic Period with Glucagon-Like Peptide-1 or Exendin-4

B. PORTHA[a], C. TOURREL[a,b], D. BAILBE[a], M. LACORNE[a], M. J. MEILE[a] and M. KERGOAT[b]

[a]*Lab. Physiopathol. Nutrition—CNRS UMR 7059,* [b]*MERCK-LIPHA, Paris, France*

In the Goto-Kakizaki (GK) rat, a genetic model of NIDDM, the neonatal β-cell mass deficit is considered to be the primary defect leading to basal hyperglycemia which is detectable for the first time 3 weeks after birth.

We have investigated in GK females the short and the long term effect of a treatment with GLP-1 or its long acting analog exendin-4, during the first postnatal week (during the pre-diabetic period). GK rats were treated with daily injection of GLP-1 (400 $\mu g \cdot kg^{-1}$) or exendin-4 (3 $\mu g \cdot kg^{-1}$) from day 2 to day 6 after birth (GK/GLP-1 and GK/Ex-4 rats) and were evaluated against Wistar and untreated GK rats. Under these conditions, both treatments enhanced, on day 7, pancreatic insulin contents and total β-cell mass by stimulating β-cell neogenesis and β-cell regeneration. Follow up of biological characteristics from day 7 to adult age (2 months) showed that such a GLP-1 or exendin-4 treatment exerted long-term favorable influence on β-cell mass and glycemic control at adult age. As compared to untreated GK rats, 2-month-old GK/GLP-1 and GK/Ex-4 rats exhibited significantly decreased basal plasma glucose. Their glucose-stimulated insulin secretion, *in vivo* after intravenous glucose load or *in vitro* using perfused pancreas, were improved. Moreover, plasma glucose disappearance rate was increased in both treated GK groups compared to untreated GK group.

These findings in the GK model of NIDDM indicate that a GLP-1 or exendin-4 treatment limited to the pre-diabetic period, delays the installation and limits the severity of NIDDM. Under these conditions, GLP-1 represents a unique tool through its β-cell replenishing effect in spontaneously diabetic rodents. It may prove invaluable agent for prevention of human NIDDM.

### Antihyperglycemic Action of Orexin-A and -B in Fasting Streptozotocin-Diabetic Mice

IKUKO KIMURA[a], YOSHITAKA SUGIHARA[a], RITSU HONDA[a],
TOSHIYASU SASAOKA[a] and TSUTOMU WADA[b]

[a]*Department of Clinical Pharmacology, Graduate School of Pharmacological Science,*
[b]*First Int. Med., Toyama Med. and Pharm. University, Toyama 930-0194, Japan*

Orexin-A and -B, neuropeptides implicated in feeding and drinking behavior and the control of sleep, have amino-acid sequences partly in common with salivatin which has an antihyperglycemic effects as a result of releasing insulin from the pancreas (Kimura, M., *et al.*, 1995). Streptozotocin (STZ)-diabetic ddY male mice (8 weeks old, 4 weeks after iv bolus injection of STZ with 150 mg/kg, blood glucose level (BGL) more than 200 mg/mL) were used after 10- to 14-hr starvation. Orexin-A and -B lowered BGL markedly 2–6 hr, after iv (10 pmol and 1 nmol/kg) and after intracerebroventricular (icv) (2 and 200 fmol/mouse) administration. The peptides did not lower the BGL in non-fasting STZ-diabetic mice or in fasting normal mice, and did not release basal insulin in fasting STZ-diabetic mice. Orexin B (10 nM) did not affect glucose (8.3 mM)-stimulated insulin release by the isolated perfused pancreas of normal rat. Orexin-A (30 nM) did not affect insulin-stimulated glucose uptake in 3T3-L1 adiopocytes. These results demonstrate that orexins lower BGL through central pathways and then stimulate food consumption to sustain normal BGL. Orexin-induced hyperphagia may be a rebound phenomenon to compensate for its anti-hyperglycemic action.

### The Effect of Leptin of Appetite and Body Temperature in Spontaneous Diabetic Nagoya-Shibata-Yasuda (NSY) Mouse

KOJI NOJIMA[a], HIROSHI IKEGAMI[a], TOMOMI FUJISAWA[a], KAZUNORI YAMADA[a],
MICHIKO ITOI-BABAYA[a], NARU BABAYA[a], MASAO SHIBATA[b] and TOSHIO OGIHARA[a]

[a]*Department of Geriatric Medicine and Osaka University Graduate School of Medicine, Osaka, Japan,*
[b]*Department of Health, Aichi-Gakuin University, College of General Education, Aichi, Japan*

To assess a role of leptin in the pathogenesis of abdominal obesity and type 2 diabetes, we investigated serum leptin levels and related phenotypes in Nagoya-Shibata-Yasuda (NSY) mouse, an animal model for type 2 diabetes. Fasting serum leptin levels at 12 weeks of age in NSY mouse were markedly higher than those of C3H mouse ($p <$ 0.0001). The food intake in the following 24 hours, however, was similar between the two strains. At 16 weeks of age, longitudinal serum leptin levels in a day (0:00, 6:00, 18:00, 24:00) were not correlated with rectal temperatures in NSY mouse. These data indicated that NSY mouse is resistant to leptin in terms of regulation of appetite and body temperature, which may contribute to abdominal obesity in this model.

### Prevention and Reversal of Nutritionally Induced Diabetes in *Psammomys Obesus*

EHUD ZIV, RONY KALMAN and ELEAZAR SHAFRIR

*Diabetes Research Unit, Hadassah University Hospital, Jerusalem, Israel*

Psammomys obesus (sand rat) is a desert gerbil maintaining normoinsulinemia and normoglycemia on a lower energy (LE) diet. When transferred to high energy (HE) diet, they become hyperinsulinemic, hyperglycemic and obese. This nutritional diabetes can be reversed by return to LE diet. Several treatments can prevent or delay the development of diabetes on HE diet. Oral administration of a vanadium salt complexed with L-glutamic acid($\gamma$)monohydoxamate (32 mg/kg, 7 days) prevents the onset of diabetes. I.P. injection of vanadyl sulfate (5 mg/kg, 5 days) to diabetes *Psammomys* also results in prolonged restoration of normoglycemia and normoinsulinemia, normalization of glucose tolerance and reduction in hepatic phosphoenolpyruvate carboxykinase activity. Another effective antidiabetic treatment is addition of 2.3% of Fenu-Greek seed extract ("Fenu-Pure") to HE diet. The preventive and hypoglycemic effects of vanadium salts and of Fenu-Pure were achieved without reduction in food consumption or body weight. Another method ameliorating the diabetic state is electroacupuncture at special abdominal acupoints. Blood glucose levels in diabetic Psammomys return to normal for more than 3 weeks without weight loss. There are several possible mechanisms for the above effects. Vanadium salts potentiate insulin action and activate the signaling by insulin leading to GLUT4 translocation. Electroacupuncture also increases insulin sensitivity. Fenu-Pure acts on food ingestion—lowering the glycemic food index. All these treatments emphasize the complexity of the diabetic state in *Psammomys* and have implications for treatment of certain human populations, for which the *Psammomys* is an excellent model of disease progression.

### High Fat Feeding in the Göttingen Minipig Impairs Fasting Glucose and Marginally Decreases Insulin Sensitivity

M. O. LARSEN, M. WILKEN, R. D. CARR and B. ROLIN

*Novo Nordisk A/S and the Royal Veterinary and Agricultural University, Denmark*

High fat feeding can induce glucose intolerance and insulin resistance in rodents and is an important aspect for establishing animal models of clinical type 2 diabetes because dietary fat and obesity are associated with the development of the disease. The present study was performed to investigate the effects of relatively short term high fat feeding in male Göttingen minipigs 7–8 months of age randomised to receive either control (C) ($n = 5$) (5% fat) or high fat (HF) diet ($n = 4$) (20% fat). Food intake was adjusted to keep C group normal weight and HF group 50% overweight. After 3 months of diet feeding animals were scanned for body composition and an oral glucose tolerance test (2 g/kg glucose) was performed measuring plasma glucose (PG) and insulin (PI). Results are summarised in Table 1. In conclusion, 3 months of HF feeding resulted in moderate obesity, a doubling of body fat and increased fasting glucose whereas oral glucose tolerance was not significantly affected. However, the increases in both fasting and post-prandial insulin levels indicated development of increased insulin resistance. Therefore, further investigation of high fat fed minipigs may provide a useful, large animal model of type 2 diabetes.

**Table 1**
**Characteristics of C and HF Fed Animals after 3 Months of Diet Feeding**

| Group | Body Weight kg | Body Fat % | Fasting PG mM | Fasting PI mM | AUC PG mMxmin | AUC PI pMxmin |
|---|---|---|---|---|---|---|
| C | 24.9 ± 0.5 | 6.1 ± 0.5 | 3.6 ± 0.3 | 23 ± 21 | 801 ± 141 | 18958 ± 3952 |
| HF | 32.6 ± 2.4 | 13.2 ± 3.2 | 4.3 ± 0.4 | 80 ± 23 | 923 ± 98 | 35523 ± 21969 |
| $P_{value}$ | 0.001 | 0.002 | 0.023 | 0.012 | 0.192 | 0.137 |

### Characterisation of Selectively Bred Diet Induced Obese Rats as a Model of Impaired Glucose Tolerance and Insulin Resistance

THÓRA B. BÖDVARSDÓTTIR[a], HANNE B. JENSEN-HOLM[a], KIRSTEN RASMUSSEN[a], CHRISTINA BJENNING[a] and BARRY E. LEVIN[b]

[a]*Pharmacological Research I, Pharmacology, Diabetes Discovery, Novo Nordisk, Bagsvaerd, Denmark,*
[b]*Neurol. Svc [127C], VA Medical Center, East Orange, NJ, USA*

Rodent models commonly used in the quest for treatment of type 2 diabetes often exhibit a single gene defect which is not the case with most human subjects. For this reason, we used a polygenic model of rats selectively bred to develop diet-induced obesity (DIO) associated with impaired glucose tolerance, dyslipidemia and insulin resistance when fed a high-fat diet to test the hypothesis that these conditions could be reversed by treatment with a known insulin-sensitiser (Rosiglitazone). Low fat (chow)-fed DIO rats ($n$ = 12) underwent an oral glucose tolerance test (OGTT) where glucose and insulin was measured as well as fasting triglyceride (TG), free fatty acid (FFA) and glycosylated haemoglobin ($HbA_{1C}$) levels. The rats were then transferred to a high-fat (32%) diet and the OGTT was repeated at 10 and 24 days. The rats were then allocated into two groups randomised to have the same mean area under the glucose curve. One group received Rosiglitazone 10 mg/kg, once daily for 8 days and the other group vehicle. High-fat diet intake for 10 to 24 days increased fasting blood glucose, insulin, TG, FFA and $HbA_{1C}$ levels associated with decreased glucose tolerance and insulin sensitivity. Rosiglitazone treatment for 8 days of these glucose intolerant, dyslipidemic and insulin resistant rats reduced fasting blood glucose, insulin, TG, FFA and $HbA_{1C}$ levels and increased glucose tolerance and insulin sensitivity to control levels. The rats receiving Rosiglitazone had the same caloric intake as the vehicle group, but gained significantly more weight, thus having a higher feed efficacy.

In summary, Rosiglitazone is an effective insulin-sensitising drug when tested in a polygenic model of DIO with insulin resistance and dyslipidemia. This model should prove useful for investigation of the pathology and treatment of type 2 diabetes.

### Calpain 3 Gene Expression in Skeletal Muscle Is Associated with Body Fat Content and Measures of Insulin Resistance

K. WALDER[a], J. MCMILLAN[a], N. LAPSYS[b], A. KRIKETOS[b], A. CIVITARESE[a], A. SOUTHON[a], P. ZIMMET[c] and G. COLLIER[a]

[a]*Metabolic Research Unit, Deakin University, Australia,* [b]*Garvan Institute of Medical Research, Sydney, Australia,* [c]*International Diabetes Institute, Caulfield, Australia*

A genetic variant in *CAPN10*, which affects its expression levels, was recently implicated in the regulation of carbohydrate oxidation and the development of Type 2 diabetes. We hypothesised that variation in skeletal muscle expression of *CAPN3*, the gene encoding the muscle-specific calpain, may also be associated with insulin resistance. Expression of *CAPN3* in skeletal muscle biopsies was measured using Taqman PCR in 27 non-diabetic human subjects for whom body composition was assessed by DEXA and glucose tolerance was measured by euglycemic-hyperinsulinemic clamp, and in muscle samples obtained from *Psammomys obesus*, a polygenic animal model of obesity and Type 2 diabetes. In human subjects, calpain 3 gene expression was negatively correlated with total ($p = 0.022$) and central abdominal fat mass ($p = 0.034$), and with blood glucose concentration in non-obese subjects ($p = 0.017$). In *Psammomys obesus*, calpain 3 gene expression was negatively correlated with glucose ($p = 0.013$) and insulin ($p = 0.034$), and with body fat mass ($p = 0.049$). Indirect calorimetry revealed associations between calpain 3 gene expression and carbohydrate oxidation ($p = 0.009$) and energy expenditure ($p = 0.013$). Lower levels of expression of calpain 3 in skeletal muscle are associated with reduced carbohydrate oxidation and elevated circulating glucose and insulin concentrations, and also with increased body fat, and in particular abdominal fat. Therefore, reduced expression of calpain 3 in both humans and *Psammomys obesus* is associated with phenotypes related to obesity and insulin resistance.

### Effect of IL-1 Secreted after Moderate Exercise on Blood Glucose Level in Type 2 Diabetes Mice and Non Diabetic Mice

AKIRA TAMAGAWA and JO SATOH

*School of Medicine of Tohoku University, Sendai, Japan*

Mouse strain KK-Ay is a model of type 2 diabetes. A single bout of exercise improves the glucose tolerance of KK-Ay mouse, which implies that a distinct mechanism other than insulin is involved in the improvement. Our previous data indicate that IL-1 reduces the level of blood glucose. So we hypothesized that IL-1 might be involved in the improvement of glucose tolerance after exercise. Male KK-Ay or C3H/HeN mice were exercised for 1 hr. After exercise, 10 ml of 20% glucose solution/kg body weight was injected ip (GTT). Blood glucose was immediately measured. Serum insulin and IL-1 concentrations were measured by ELISA. The peak blood glucose level after GTT was significantly reduced in the exercise group in comparison with the sedentary groups. The improvement of glucose tolerance observed after exercise was reversed by the treatment of anti-IL-1 alpha antibody. Though this result was identical for both KK-Ay and C3H/HeN strains, the improved glucose tolerance in C3H/HeN was remarkable compared to KK-Ay mice. Furthermore, the level of IL-1 alpha secreted after the moderate exercise was markedly higher in C3H/HeN mice than in KK-Ay mice. We concluded that IL-1 is involved in the reduction of blood glucose level after exercise in both group of mice. Furthermore, it was suggested that reduced secretion of IL-1 alpha might be involved in the pathophysiology of type 2 diabetes.

### β-Cell Neogenesis from Duct Cells in Partial Duct-Ligated Mice

M. LI[a], K. YAMAMOTO[b], M. MORIWAKI[a], M. YEN[a], T. NAMMO[a], A. IMAGAWA[a], H. IWAHASHI[a], K. YAMAGATA[a], J. MIYAGAWA[a], and Y. MATSUZAWA[a]

[a]*Department of Internal Medicine and Molecular Science, Graduate School of Medicine, Osaka University,*
[b]*Department of General Medicine, Osaka University Hospital, Suita, Japan*

*[Aim]* To clarify the mechanism of islet cell neogenesis from duct cells, morphological changes in the pancreatic duct-ligated mice were analyzed. *[Method]* The pancreatic ducts of the splenic lobe were ligated in 8 wk-old male ICR mice. At day 3, 7, 14 after the operation, the pancreas was removed and processed for immunohistochemistry. Double immunostaining for BrdU and duct cell-specific cytokeratin (DCK) were performed to calculate BrdU labeling index (BrdU LI) of duct cells. The pancreatic hormones and several kinds of transcriptional factors that are related to islet cell differentiation including IPF1/PDX1 were doubly immunostained. *[Results]* At day 1, the serum amylase level was highest and features of acute pancreatitis could be histologically detected, while glucose intolerance was not recognized after duct ligation. From day 3, the BrdU LI of duct cells in ligated portion was increased compared to non-ligated portion or control. From day 7, the glucagon or insulin positive cells were observed in ducts. The IPF1/PDX1-positive cells were also observed in duct cells. *[Discussion]* In this model, β-cell neogenesis from ducts was detected and IPF1/PDX1 was shown to be essential for β-cell neogenesis even without presence of glucose intolerance.

### Normalization of Hyperglycemia after Adenovirus-Mediated Gene Transfer of IRS-2 into IRS-2 Deficient Mice

MASASHI AOYAMA[a], KAZUYUKI TOBE[a], RYO SUZUKI[a], YASUO TERAUCHI[a], NAOTO KUBOTA[a], KAZUHIRO ETO[a], KAJURO KOMEDA[b] and TAKASHI KADOWAKI[a]

[a]*Department of Diabetes and Metabolic Diseases, University of Tokyo, Graduate School of Medicine, Bunkyo-ku, Tokyo, Japan,*
[b]*Animal Research Center, Tokyo Medical University, Shinjyuku-ku, Tokyo, Japan*

IRS-2–/– mice progressively developed type 2 diabetes due to both insulin resistance and relative insulin deficiency associated with decreased b-cell mass in the face of insulin resistance. We have explored the mechanism of insulin resistance in IRS-2–/– mice and attempted to treat diabetes by adenovirus-mediated gene therapy. We infected the adenovirus encoding IRS-2 and LacZ into diabetic IRS-2–/– mice at 10 weeks intraperitoneally and compared them with uninfected wild type mice. IRS-2 in liver of adeno-IRS-2 infected IRS-2–/– mice was expressed to almost the same level as in liver of wild type mice, where IRS-2 expression in skeletal muscle or adipose tissue was not detectable. A glucose load test 3 day after infection revealed that adeno-IRS-2-infected IRS-2–/– mice showed normal glucose tolerance while LacZ-infected mice remained diabetic. Insulin tolerance test

(ITT) showed that restoration of IRS-2 in the live of diabetic IRS-2–/– mice by infecting adeno-IRS-2 resulted in the increased sensitivity to the almost same level as the wild type mice, suggesting that insulin resistance in IRS-2–/– mice was due to the defects in insulin signaling in liver. These data also suggested that both insulin resistance and defects in insulin secretion are required for the development of type 2 diabetes.

### Dysfunction of GIP/Insular Axis as Pathogenesis of Diabetes

K. TSUKIYAMA, K. MIYAWAKI, Y. YAMADA and Y. SEINO

*Department of Metabolism and Clinical Nutrition, Graduate School of Medicine, Kyoto University, 54 Shogoin-Kawahara-cho, Sakyo-ku, Kyoto 606-8507, Japan*

To analyze the role of gastric inhibitory polypeptide (GIP) as a mediator of signals from the gut to the pancreatic β-cells, we have generated mice with a targeted mutation of the GIP receptor (GIPR) gene. *GIPR*-deficient mice showed higher blood glucose levels accompanied with impaired initial insulin response after oral glucose loading. The maximum blood glucose levels of *GIPR*-deficient mice were significantly higher than those of wild-type mice. These demonstrate that insulin secretion, especially in the early phase after oral glucose loading, is mediated by GIP, and that an intestinal factor contributes to development of diabetes. To elucidate the interplay of the intestinal factor with insulin resistance and impaired insulin secretion for the development of type 2 diabetes, we generated mice with disruption of *GIPR* and insulin receptor substrate-1 (IRS-1) or Kir 6.2 genes, a key protein of insulin signaling pathway or insulin secretory mechanism, respectively. Each double knockout mouse showed more progressive glucose intolerance than that of single *Kir 6.2* or *IRS-1* knockout mouse. In oral glucose tolerance test, the maximum blood glucose levels were 341 ± 19 mg/dl at 30 min (*kir 6.2* knockout), 407 ± 17 mg/dl at 60 min (*kir 6.2, GIPR* double knockout), 452 ± 8 mg/dl at 30 min (*IRS-1* knockout), 527 ± 39 mg/dl at 30 min (*IRS-1, GIPR* double knockout). In conclusion, GIP has an important role in development of diabetes as a factor of polygenic disorder with insulin resistance or impaired insulin secretion.

### AICAR-Treatment Improves Glucose Homeostasis in Insulin Resistant Diabetic (*ob/ob*) Mice

XIAO MEI SONG, MAJ FIEDLER, DANA GALUSKA, JEFFREY RYDER, MARIA FERNSTRÖM, KJELL S. SAKARIASSEN, YIN LIANG, MEI YU, ALEXANDAR CHIBALIN, ANNA KROOK, HARRIET WALLBERG-HENRIKSSON and JULEEN R. ZIERATH

[a]*Department of Physiology and Pharmacology, Karolinska Institutet, SE-171 77 Stockholm, Sweden,*
[b]*Department of Clinical Physiology, Karolinska Hospital, SE-171 76 Stockholm, Sweden,*
[c]*Pharmacia Corporation, Department of Lead Pharmacology SE-751 82, Uppsala, Sweden*

AMP-activated protein kinase (AMPK) has been implicated as an important mediator of muscle contraction-induced glucose transport and a target for pharmacological intervention to treat Type II diabetes and obesity. AMPK can be artificially activated by 5-aminoimidazole-4-carboxamide ribonucleoside (AICAR). We hypothesized AICAR may be efficacious in the treatment of diabetic *ob/ob* mice. An acute AICAR injection (1 mg/g body wt) normalized glucose levels in *ob/ob* mice within 1 hr, with effects persisting over 4 hr. After 1 wk of daily injections, AICAR-treatment corrected hyperglycemia, improved glucose tolerance, and increased GLUT4 and hexokinase II expression in skeletal muscle, but had deleterious effects on plasma FFA and triglycerides. AICAR treatment increased liver glycogen in fasted and fed *ob/ob* mice and muscle glycogen in fasted, but not fed *ob/ob* and lean mice. Defects in insulin-stimulated PI 3-kinase and glucose transport in skeletal muscle from *ob/ob* mice were not corrected by AICAR treatment. Importantly, while *ex vivo* insulin-stimulated glucose transport was reduced in isolated muscle from *ob/ob* mice, the AICAR-stimulated response was normal. In conclusion, AICAR-mediated improvements in glucose homeostasis in *ob/ob* mice cannot be entirely explained by effects in skeletal muscle. Due to the apparent deleterious effects of AICAR on the blood lipid profile, strategies to develop tissue- and pathway-specific activators of AMPK should be considered to improve glucose homeostasis in diabetes.

### Glucose Absorption from Small Intestine in GK Rats: Study with the Isolated Perfused Intestine

K. TANAGAWA, E. ISHIHARA, Y. ITO and T. MIURA

*Department of Clinical Nutrition, Suzuka University of Medical Science, Mie, Japan*

We studied *in vitro* glucose absorption from small intestine by means of the isolated perfusion in GK rats, an animal model of type 2 diabetes mellitus. 10 to 3 wk GK and Wistar rats were fasted for 24 hr and a perfusion was performed according to the method described by Levin *et al.* The superior mesenteric artery was perfused with KRB containing either 60, 140 or 300 mg/dl glucose. 20 min following vascular perfusion, luminal perfusion started with 3% glucose solution for 30 min. Glucose absorption was estimated from the glucose concentrations in the portal venous effluent. Integrated glucose absorption was significantly decreased in Wistar rats, when glucose concentrations in the arterial perfusate were raised to 140 and 300 mg/dl. On the other hand, no change was found in GK rats. Therefore, glucose absorption from the small intestine was greater in GK rats than in Wistar rats. These data suggest that elevated blood glucose levels in the spontaneously diabetic GK rats are, at least partially, attributed to increased glucose absorption from the small intestine.

### IGF-1 as Target Antigen in Type 1 Diabetes

KEIICHI KODAMA, AKIRA SHIMADA, OSAMU FUNAE, JIRO MORIMOTO, MIKIYA TOKUI, KENJI WATANABE and TAKAO SARUTA

*Keio University School of Medicine, Tokyo, Japan*

It has previously been suggested that target antigens of the autoimmune process in type 1 diabetes may be "proteins in islets" such as GAD or (pro)insulin, although this issue is still controversial. Here we report significance of "a protein out of islets," IGF-1, as a target antigen, which is mainly produced in liver and has homology to insulin. NOD (female) mice were immunized with subcutaneous injection of IGF-1, GAD or insulin in IFA or IFA only at 3–4 weeks of age. Diabetes onset was significantly suppressed in IGF-1 group as compared to GAD ($p < 0.05$), insulin ($p < 0.05$), and control group ($p < 0.05$). Moreover, diabetes onset was delayed in IGF-1 group as compared to GAD ($p < 0.02$) and control group ($p = 0.052$). There was no significant difference in degree of insulitis between IGF-1 and control group. IGF-1 group showed significantly lower IFN-γ production upon polyclonal stimulation than control ($p < 0.01$). In conclusion, it has been suggested that IGF-1 immunization but not GAD or insulin suppressed diabetes in NOD mice. Immunizing NOD mice with several IGF-1 peptides to detect major T cell epitopes of IGF-1 is in progress.

### Contribution of Fas/FasL Interaction in the Initial Phase of NOD Autoimmune Diabetes

M. NAKAYAMA, M. NAGATA, H. YASUDA, R. KOTANI, K. YAMADA, S. A. CHOWDHURY, K. YOKONO and M. KASUGA

*Kobe University School of Medicine, Japan*

Fas/FasL interaction plays a crucial role in the development of autoimmune diabetes. However, it was reported that blockade of Fas/FasL interaction in the effector phase was not enough to prevent diabetes. In this study, we elucidate the role of Fas/FasL interaction in the initial phase of NOD diabetes.

When NOD mice were administrated with anti-FasL antibody (MFL1) from 2 to 4 wk of age, neither diabetes nor insulitis was observed. In addition, splenocytes from MFL1-treated NOD mice failed to transfer diabetes into NOD-scid mice. Although no effector activity was observed, MFL1-treated NOD T cells were stimulated and secreted IFN-gamma in response to islet cell antigen. To investigate whether MFL1-treatment leads to APC dysfunction, diabetic splenocytes were injected into MFL1-treated NOD mice. Although transfer of whole diabetic splenocytes reversed overt diabetes in MFL1-treated NOD, diabetic T cells or non-T cells could not transfer diabetes. These results suggest that MFL1-treatment at young age induced APC dysfunction and caused unknown qualitative change of autoreactive T cells.

In conclusion, Fas/FasL interaction plays a crucial role in the initial phase of autoimmune diabetes, which influenced the development of autoreative T cells and APC.

### Blockade of CD30/CD30L Interaction Leading to Suppression of NOD Diabetes

CHAKRABARTY SAGARIKA, NAGATA MASAO, NAKAYAMA MAKI, CHOWDHURY SHAHEAD ALI, YASUDA HISAFUMI, YAMADA KATSUMI, KIN TEIKO, FUJIHIRA KAZUHIRO and YOKONO KOICHI

*Department of Geriatric Medicine, Kobe University School of Medicine*

CD30/CD30L is a member of TNF/TNFR superfamily and has been shown to play a critical role in immunoregulation, suggested by the fact that CD30-deleted CD8T cells were facilitated to destroy pancreatic islets. Here we characterized the expression of CD30/CD30L and tried functional blocking of CD30/CD30L interaction in NOD mice by using a monoclonal antibody against CD30L(RM153).

The expression of CD30/CD30L on αCD3-activated NOD splenic T cells were evaluated by Flow cytometric analysis. The result showed the expression of CD30 primarily on CD8T cells and CD30L on CD4T cells as well as control mice. Four-week-old female NOD mice were injected with 500 μg RM153 twice a week up to 10 weeks of age, and then evaluated the incidence of diabetes and insulitis at 30 weeks of age. Only 36.4% of treated mice became diabetic at 30 weeks. Whereas 75% of untreated control mice became diabetic. Mild insulitis score also have been shown to RM153 treated mice. $4 \times 10^6$ islet derived CD8+CTL isolated from 15–18-wk-old female NOD islets were injected in 7 day old female NOD mice with or without treatment of 500 μg RM153. All the control recipients developed diabetes by 2 weeks. However, none of RM153 treated NOD recipients have shown diabetes until 5 weeks.

These results suggest that CD30/CD30L interaction is not contributed to negative immunoregulation against autoimmune diabetes in NOD mice. From our observation, blocking of CD30/CD30L interactions did not facilitate rather delayed development of insulitis and diabetes.

### Hematopoietic Cell Transplantation for Autoimmune Diseases: Studies in NOD Mice

GEORG F. BEILHACK, MARILYN A. MASEK and JUDY A. SHIZURU

*Stanford University, USA*

It has been shown that transplantation of MHC-mismatched allogeneic whole bone marrow cells can block the pathogenesis of autoimmune diseases. However, little data exist that demonstrate the effects of clinically relevant hematopoietic cell grafts on autoimmune disease outcome. These grafts include autologous/syngeneic, haploidentical and MHC-matched (mHC mismatched) sources. The goal of our studies is to find the optimal graft composition for hematopoietic cell transplantation (HCT), which blocks the autoimmune process and prohibits recurrence of disease after transplantation with limited morbidity and mortality.

In prior studies we showed that transplantation of MHC-mismatched purified hematopoietic stem cells from AKR/J mice prevents the onset of autoimmune diabetes in prediabetic non-obese diabetic (NOD) mice. Here, we show that HCT of (pseudo)autologous NOD.Thy1.1 and haploidentical donor cells (NODxAKR)F1 do not block the onset of diabetes in prediabetic NOD mice, whereas transplantation of MHC-matched unrelated donor hematopoietic cells, C57BL/6.H-$2^{g7}$ confer protection. These findings are surprising given that the (NODxAKR)F1 cells express (at a one gene dose) a presumably protective MHC allele, whereas cells from the C57BL/6.H-$2^{g7}$ donors express the disease associated MHC allele. The data suggest non-MHC background genes expressed in donor derived hematopoietic cells can block autoimmune disease pathogenesis. The human correlates of these studies are matched and unrelated donor transplants, which are routinely performed in patients with malignancies and bone marrow failure states.

### Prevention of Diabetes Development by 5 α-Dihydrotestosterone in Female NOD Mice

S. TAKEI, T. AWATA, S. KATAYAMA

*Fourth Department of Int. Med., Saitama Medical School, Saitama, 350-0495, Japan*

Nonobese diabetic (NOD) mice have been utilized as a model for human type 1 diabetes. The incidences of diabetes in female and male NOD mice are 65% and 5% at 30 weeks of age, respectively. This sexual dimorphism suggests that sex-steroid hormones may play an important role in the development of the disease. We already reported that a male stëroid, 5 α-dihydrotestosterone (5DHT), has a potential to prevent the development of insulitis as well as diabetes in the female NOD mice. Results also demonstrated that 5DHT has direct systemic effects on the expansion of Th2 cell populations with subsequent restoration of normal immune responses. However, it is unclear regarding a critical time point on systemic 5DHT administration which leads to preventing the disease development. Thus, in this study we examined a critical time point on 5DHT administration for the suppression of disease development. We also examined whether 5DHT administration was capable of restoring IL-4 expression even in inflamed islets. *In vitro* administration of 5DHT to splenocytes isolated from 4-week female NOD mice demonstrated an increased expression of IL-4 gene representing Th2 cell populations compared with age-matched controls. Whereas that to splenocytes isolated from 9-week mice did not increase the expression of IL-4 gene. Furthermore, *in vitro* administration of 5DHT to inflamed islets isolated from 14-week females demonstrated an increased expression of IL-4 gene compared with age-matched controls. These results demonstrate that 5DHT administration at 4-week (pre-insulitis stage) is a critical time point to prevent the development of diabetes. Results also demonstrate that 5DHT administration affects systemic as well as localized immune responses.

### Genome Analysis of the Rat Autoimmune Type 1 Diabetes Locus *Iddm/kdp1*

NORIHIDE YOKOI[a], KAZUKI YASUDA[a], KAJURO KOMEDA[b], TADAO SERIKAWA[c] and SUSUMU SEINO[a]

*[a]Chiba University, Chiba, Japan, [b]Tokyo Medical University, Tokyo, Japan, [c]Kyoto University, Kyoto, Japan*

The Komeda diabetes-prone (KDP) rat is an animal model for human type 1 diabetes. We have mapped a major diabetes locus, termed *Iddm/kdp1*, on rat chromosome 11, and also constructed a physical map of the region in the mouse genome. Here, we performed physical mapping of the region in the rat genome, and also conducted genome analysis in the mouse genome. The rat YAC and BAC libraries were screened with SSLP and STS markers flanking the region, the mouse BAC clones were sequenced and homology search was performed. As a result, the *Iddm/kdp1* region was covered with the rat YAC/BAC contigs, and it is revealed that there are several transcript units in the region. These putative transcript units would be potential candidates for the *Iddm/kdp1*. We are currently searching the mutation involved in the pathogenesis on type 1 diabetes in the KDP rat.

### Age-Dependent Effect of a QTL in Spontaneous Diabetes Model, Nagoya-Shibata-Yasuda (NSY) Mouse

MICHIKO ITOI-BABAYA[a], HIROSHI IKEGAMI[a], HIRONORI UEDA[a], YOSHIHIKO KAWAGUCHI[a], TOMOMI FUJISAWA[a], KOJI NOJIMA[a], KAZUNORI YAMADA[a], NARU BABAYA[a], MASAO SHIBATA[b] and TOSHIO OGIHARA[a]

[a]*Department of Geriatric Medicine, Osaka University Graduate School of Medicine, Osaka, Japan,*
[b]*Department of Health, Aichi-Gakuin University, College of General Education, Aichi, Japan*

To assess a gene-environmental interaction in the dependent of type 2 diabetes, the F2 progeny between spontaneously diabetic Nagoya-Shibata-Yasuda (NSY) mouse and non-diabetic C3H mouse were subjected to genetic analysis using markers on chromosome 1, where a suggestive quantitative trait locus (QTL) was detected in the initial screening. Significant evidence of linkage was found in the region near *D1Mit14*, with maximum LOD score (MLS) of 6.3 for area under the glucose curve (AUC) after intraperitoneal glucose challenge, at 12 weeks of age. This locus had suggestive linkage to AUC at 24 and 36 weeks of age (MLS: 3.5 and 2.8, respectively), but no evidence of linkage at 48 weeks of age (MLS: 2.0). No linkage was found to body weight, plasma insulin, body mass index or fat-pad weight. These data demonstrate that a QTL for type 2 diabetes on chromosome 1 in the NSY mouse is age-dependent, indicating the importance of considering age-dependent changes in phenotypes in the genetic analysis of polygenic traits, such as type 2 diabetes.

### Genetic Dissection of Type 2 Diabetes Using Consomic Strains Carrying Diabetogenic Chromosomes 11 and 14 from Spontaneously Diabetic NSY Mice

NARU BABAYA[a], HIROSHI IKEGAMI[a], YOSHIHIKO KAWAGUCHI[a], TOMOMI FUJISAWA[a], HIRONORI UEDA[a], KOJI NOJIMA[a], MICHIKO ITOI-BABAYA[a], KAZUNORI YAMADA[a], MASAO SHIBATA[b] and TOSHIO OGIHARA[a]

[a]*Department of Geriatric Medicine, Osaka University Graduate School of Medicine, Osaka, Japan,*
[b]*Department of Health, Aichi-Gakuin University, Aichi, Japan*

Our previous whole genome linkage study of Nagoya-Shibata-Yasuda (NSY) mouse, a spontaneous model for type 2 diabetes, showed significant evidence for linkage for glucose intolerance on chromosomes 11 and 14. We established two consomic strains, C3H.NSY-chr11 and C3H.NSY-chr14, in which a whole diabetogenic chromosome (11 or 14) was introgressed onto the control genetic background (C3H). The C3H.NSY-chr11 mice exhibited significantly higher blood glucose and fasting insulin levels and lower insulin secretion after a glucose challenge that C3H mice. C3H.NSY-chr14 mice showed significantly higher fasting insulin level, whereas they showed similar blood glucose level and insulin secretion to C3H mice. Body weight was similar among the three lines. These data suggest that diabetogenic chromosome 11 of the NSY mouse affects glucose tolerance through both insulin resistance and insulin secretion, and that the diabetogenic chromosome 14 causes insulin resistance, which does not cause glucose intolerance in itself. Consomic/congenic strategy makes it feasible to dissect a complex trait into each genetic component.

### Linkage Analysis of Responsible Loci for Glucose Intolerance in Non-Obese Spontaneously Diabetic Torii (SDT) Rat

T. MASUYAMA[a], M. FUSE[b,c], A. HARA[b,c], M. SUGAWARA[b], M. KANAZAWA[c], Y. KANAZAWA[d] and K. KOMEDA[c]

[a]*Torii Pharmaceutical Co.,* [b]*Nippon Veterinary and Animal Science University,* [c]*Tokyo Medical University, Tokyo, Japan,* [d]*Jichi Medical School, Saitama, Japan*

The spontaneously diabetic Torii (SDT) rat, characterized by glucose intolerance after 16 weeks of age, hyperglycemia and glycosuria after 20 weeks of age, and fibrosis of pancreatic islets by 25 weeks of age in the males, is a new animal model for studies of type 2 diabetes mellitus in humans (*Int. J. Exp. Diab. Res.,* 1:89–100, 2000). We performed pathological and physiological characterizations and genetic linkage analysis using a backcross panel [(BN × SDT) F1 × SDT]. All of the F1 progenies showed no sign of glucose intolerance at 20 weeks of age and hyperglycemia at 25 weeks of age in the males. Only 27 rats (8.5%) out of 319 male backcrosses showed glucose intolerance at 20 weeks of age or hyperglycemia at 25 weeks of age. Pancreatic islets from the 27 rats were observed to undergo pathological changes such as fibrosis and decrease in the number of β cells. By the first genome-wide scan for responsible genes in this strain using SSLP markers, we mapped five loci for glucose intolerance; these were *Nidd/sdt1, Nidd/sdt2, Nidd/sdt3, Nidd/sdt4,* and *Nidd/sdt5* on rat chromosomes 1, 2, 11, 18 and X, respectively. We conclude that glucose intolerance is a polygenic trait, and distinct combinations of genetic loci are responsible for glucose intolerance in the SDT rat.

### Inhibition of Retinal Fibronectin Overexpression Prevents Capillary Basement Membrane Thickening in Galactose-Fed Rats: An Animal Model of Diabetic Retinopathy

S. ROY, T. SATO, R. KAO and G. PARYANI

*Boston University School of Medicine, Boston, MA, USA*

Increased synthesis of extracellular matrix (ECM) components including fibronectin is closely associated with the development of vascular basement membrane (BM) thickening, a prominent characteristic of diabetic microangiopathy. We investigated whether the thickening of retinal capillary basement membrane in galactose-fed rat, an animal model of diabetic retinopathy, could be prevented by down regulating fibronectin (FN) synthesis using antisense oligonucleotides (oligos) targeted against translation initiation site of the FN transcript. Sprague Dawley rats were divided into three groups: control, galactose-fed, and galactose-fed treated with antisense oligos. The latter group received intravitreal injection of 3μM fibronectin antisense oligos together with a delivery vehicle polyoxyethylene-polyspermine block copolymers at one month interval for three months. Using RT-PCR retinal fibronectin mRNA level was determined at one-, three- and seven month time points. Retinal capillary BM thickness was determined from electron micrographs representing seven month time point. Glycohemoglobin levels, monitored at two month intervals were consistently elevated in the galactose-fed rats compared to control rats (6.9 0.6% vs. 4.5 ± 0.8%, $p = 0.01$). Galactose-fed rats treated with FN-antisense oligos showed significant reduction in the retinal capillary BM thickness compared to untreated galactose-fed rats (123 ± 16 nm vs. 201 ± 12.3 nm, $p <$ 0.03). The retinal fibronectin mRNA level in rats treated with antisense FN-oligos showed reduced fibronectin expression (69 ± 7% of galactose-fed group, $p < 0.01$; $r = 0.9$). Our findings indicate that antisense oligos are effective in down regulating fibronectin overexpression and arresting retinal capillary BM thickening. The antisense oligos provide a powerful interventive strategy for studying the role of vascular BM thickening in diabetic microangiopathy.

### Evidence for the Existence for Oxidative Stress in Glomeruli from Diabetic Rats

DAISUKE KOYA, MASAKAZU HANEDA, HAYASHI KAZUYUKI, KITADA MUNEHIRO and RYUICHI KIKKAWA

*Third Division, Shiga University of Medical Science, Otsu, Shiga, Japan*

Accumulating evidence relating to the genesis of diabetic vascular complications suggests that oxidative stress can alter a variety of biological gene expression and finally results in diabetic pathologies. However, it is not yet clarified whether oxidative stress has a primary role in the development of diabetic nephropathy or is merely reflecting resultant tissue damage by diabetes. To answer this question, we examined whether the oxidative stress might occur in diabetic glomeruli at an early stage of diabetic rats by visualizing H2O2. The glomeruli from diabetic rats revealed the intense staining of dichlorofluorescein as compared to those from non-diabetic control rats. We next tried to prove the existence of oxidative stress by examining glomerular mRNA expression of a variety of antioxidant enzymes, which are induced response to oxidative stress and are implicated to protect cellular and tissue functions to keep *in vivo* homeostasis. The mRNA expression of antioxidant enzymes such as catalase (CAT), glutathione peroxidase (GPx), thioredoxin reductase (Trx), and Cu, Zn-superoxide dismutase (Cu, Zn-SOD) of diabetic glomeruli was comparable to that of non-diabetic control rats. In contrast, the mRNA expression of heme oxygenase 1 (HO-1) and Mn-SOD, but not HO-2 was increased in diabetic glomeruli as compared to that in control rats. In addition, we found that either insulin or an antioxidant treatment was able to normalize this enhanced HO-1 expression. In conclusion, we provide the evidence that oxidative stress occurs in glomeruli at an early stage of diabetes and among antioxidant enzymes HO-1 and Mn-SOD expression is preferentially increased in diabetic glomeruli.

### Altered Sensory Thresholds in Diabetic Rats

Y. KISHI[a], M. KISHI[a], H. SASAKI[a], K. NANJO[a] and P. A. LOW[b]

[a]*The First Department of Medicine, Wakayama University of Medical Science, Wakayama, Japan,*
[b]*Departments of Neurology, Mayo Foundation, Rochester MN, USA*

Sensory thresholds of diabetic rats were studied by several investigators but results have been inconsistent. We therefore undertook an evaluation using a well-characterized animals and well-standardized methodology. We evaluated thermal thresholds to noxious radiant heat and touch thresholds in streptozotocin induced diabetic rats using a Hargreave's system and Semmes-Weistein monofilaments respectively. We also evaluated changes of pain thresholds in the chronic constriction injury (CCI) model with diabetes. Decreased touch thresholds were observed in the 6-week-duration diabetic rats (controls 3.94 ± 0.29 g vs. diabetes 2.65 ± 0.32 g, $P$ value = 0.0061). CCI, peaking at day 14, caused affected limbs thermal hyperalgesia expressed as difference scores subtracting the reaction time on the non-injured paws from those on the injured paws (controls 0.06 ± 0.06 s vs. CCI –0.95 ± 0.09 s, $P$ value < 0.0001). Hyperalgesia in CCI model with diabetes was attenuated (CCI –0.95 ± 0.09 s vs. CCI with diabetes –0.66 ± 0.09 s, $P$ value = 0.0276). We concluded that diabetic rats have altered sensory thresholds and a Hargreave's system and Semmes-Weinstein monofilaments are sensitive and reliable tests to detect those changes.

### The Effect of C-Peptide on Cell Proliferation of Human Neuroblastoma Cell with and without Insulin or IGF-1

ANDERS A. F. SIMA, ZHEN-GUO LI and WEIXIAN ZHANG

*Wayne State University, Detroit, MI, USA*

The biological effects of C-peptide are not known. However, previous studies from our laboratory have indicated that C-peptide has anti-apoptotic and neuroprotective functions. In order to examine the potential mitogenic effect of C-peptide, we exposed human neuroblastoma SH-SY5Y cells to C-peptide alone or in combination with insulin and IGF-1. In this system, we monitored cell proliferation and neurite outgrowth. The increase in cell number (4 days) with C-peptide alone was: 164 ± 9 (±SD); 188 ± 6; and 172 ± 10% ($p < 0.001$) for 1.0, 3.3 and 10 nM respectively. Maximal effect with insulin alone was achieved at a dose of 4 nM (204 ± 10%; $p < 0.001$), which was not different from maximal effective C-peptide dose. The combination of maximal C-peptide and insulin doses significantly (225 ± 13%; $p < 0.05$) increased cell proliferation over that of C-peptide alone. Ten nM C-peptide tended to inhibit the effect of insulin (182 ± 11%; NS). With 1 nM IGF-1 alone, and plus 1.0, 3.3 nM and 10 nM C-peptide respectively, the increases were 262 ± 13, 273 ± 10, 285 ± 9 and 252 ± 12% ($p < 0.001$). The average length of neurite outgrowth increased with C-peptide was added to insulin ($P < 0.001$), but not when it was added to IGF-1. In order to clarify the potential mechanism by which C-peptide exerts its effect, we isolated total protein and immunoprecipitated insulin and IGF-1 receptors and examined the extent of their respective receptor tyrosine phosphorylation. C-peptide increased insulin receptor phosphorylation, but not that of the IGF-1 receptor. In conclusion, C-peptide alone has an insulin-like mitogenic effect on cell proliferation and neurite outgrowth in this *in vitro* system. In combination with insulin, there is an additive effect, which is less apparent with IGF-1. These findings are supported by an increased phosphorylation of the insulin receptor, but not of the IGF-1 receptor in the presence of C-peptide.

### Dose Dependent Prevention of Early Neuropathy in the BB/Wor Rat with Human C-Peptide

ANDERS A. F. SIMA[a], MARK YOREK[b], CHRISTOPHER R. PIERSON[a], YUICHI MURAKAWA[a], ALEXANDER BREIDENBACH[c], and WEIXIAN ZHANG[a]

*[a]Detroit, MI, USA, [b]Iowa City, IA, USA, [c]Monheim, Germany*

We have previously shown that replacement of rat II C-peptide (70 ng/kg/day) in diabetic BB/Wor rats prevents the acute nerve conduction velocity (NCV), neural $Na^+K^+$-ATPase and early structural changes. In this study diabetic BB/Wor rats were replaced with 10, 100, 500, and 1,000 μg human C-peptide per kg/day for 2 mo. Ten and 100 μg human C-peptide showed small but significant ($p < 0.1$) improvements in NCV, but not in $Na^+K^+$-ATPase, nodal or axonal changes. Five hundred and 1,000 μg human C-peptide showed significant and increasing preventative effects on NCV ($p < 0.001$), $Na^+K^+$-ATPase activity ($p < 0.05$), structural normality ($p < 0.001$) nodal changes ($p < 0.001$) and axonal degeneration ($p < 0.01$). It has been suggested that the similar effects of human and rat C-peptide is due to the fully conserved midportion of the two peptides. However, the C-terminal pentapeptide, which possesses receptor/ligand type interaction, shows the same biological activity as the entire molecule. In the present study approximately 10,000× higher concentrations of human compared to rat C-peptide was necessary to achieve the same effects. These differences may be due to partial conservation of the active C-terminal pentapeptide, which differs by three amino acids between man and rat. In conclusion human C-peptide shows a dose dependent preventative effect on rat diabetic neuropathy. We conclude that C-peptide shows cross species activity and that this may be due to conserved sequences of the active C-terminal of the molecule.

### Mechanism of Sodium Load-Induced Hypertension in Wistar Fatty Rats: Defective Dopaminergic System to Inhibit Na-K-ATPase Activity in Renal Epithelial Cells

TAKEO SATOH, HIROKI TSUCHIDA, GOROU IMAI, YOSHINORI SHIMA, GOICHI OGIMOTO and SHIGERU OWADA

*Division of Nephrology and Hypertension, Department of Internal Medicine, St. Marianna University School of Medicine, Kawasaki, 216-8511, Japan*

Obesity-related non-insulin dependent diabetes mellitus (NIDDM) is frequently accompanied by hypertension. The present study was designed to clarify this mechanism. We firstly determined the blood pressure in male Wistar fatty rats (WFR), one of the NIDDM model rats, and in Wistar lean rats (WLR) as the control, with normal (0.7% NaCl) or high (7% NaCl) salt diet. We observed no difference in systolic and mean blood pressures between WFR and WLR. WFR, however, became extremely hypertensive by the high salt diet. We next investigated the mechanism for sodium sensitivity in WFR. Although the urinary excretion of dopamine (DA), a potent natriuretic factor, which reflects the ability of renal DA production, was preserved in WFR, the sodium balance with the high salt diet was positive. Moreover, Na-K-ATPase activity in isolated proximal convoluted tubule (PCT) from WFR with normal salt diet was significantly higher than that from WLR. High salt load produced a significant decrease in Na-K-ATPase activity in WLR but not in WFR. Similarly, Na-K-ATPase activity in WLR with normal salt diet was significantly inhibited by DA ($10^{-5}$ M), but not in WFR. These results indicate that WFR has characteristic of the salt-sensitive hypertension that could be caused by the excessive sodium retention with sodium load due to a defective dopaminergic system in the kidney that fails to inhibit Na-K-ATPase activity.

### Insulin Receptor Isoform Expression in Sciatic Nerve and Dorsal Root Ganglia in Type I BB/W-, BB/W C-Peptide Treated and Type II BB/Z-Diabetic Rats

ANDERS A. F. SIMA[a], YUICHI MURAKAWA[a], WEIXIAN ZHANG[a], JOHN WAHREN[b] and CHRISTOPHER R. PIERSON[a]

*[a]Detroit, MI, USA, [b]Stockholm, Sweden*

Morphometric, clinical and functional data indicate that insulin and/or C-peptide deficiencies may be responsible for components of type I diabetic neuropathy (DN), which differs from that of type II diabetes. Nodal pathology occurs in type I but not in type II DN, and the former shows more severe axonal degeneration. The insulin receptor (IR) localizes to the axolemma of the paranode and the paranodal aspect of Schwann cells, thereby suggesting a role for IR in DN. To determine whether alterations in IR isoform expression occur in neuropathy we performed RT-PCR using primers that specifically amplify a product containing exon 11 of IR, which is present in the high affinity form but absent in the low affinity form. RT-PCR was conducted on mRNA extracted from sciatic nerve (SN) and dorsal root ganglia (DRGs) from control, type I BB/W, type I BB/W C-peptide treated and type II BB/Z diabetic rats at 2 mo. In BB/W rats the low affinity form of IR is upregulated in SN, a change that is prevented by C-peptide replacement. Total IR mRNA increased 18% in type I BB/W-rats but was corrected to an 8% increase over controls in C-peptide treated BB/W rats. Total IR mRNA was not altered in type II BB/Z rats. When RT/PCR was performed on DRGs no changes were evident in IR isoform expression across the 4 animal groups and no expression of the low affinity form was detectable. Western blotting for IR on SN parallels the findings of RT-PCR. IR expression correlated inversely with serum insulin and C-peptide levels as determined by RIA. These findings suggest that insulin and/or C-peptide deficiencies promote a relative increase in the low affinity IR, which is prevented by C-peptide replacement, suggesting that C-peptide has an insulinomimetic effect. In addition, these alterations in IR isoform expression likely occur in Schwann cells as reflected by the lack of alterations in DRGs between the 4 animal groups.

### Molecular Pathology of the Node of Ranvier in Type I Diabetic Neuropathy: A Role for Insulin and C-Peptide Deficiencies?

ANDERS A. F. SIMA[a], WEIXIAN ZHANG[a], YUICHI MURAKAWA[a], JOHN WAHREN[b] and CHRISTOPHER R. PIERSON[a]

*[a]Detroit, MI, USA, [b]Stockholm, Sweden*

C-peptide replacement in type I BB/W rats prevents the metabolic and chronic structural changes of the node of Ranvier. Nodal integrity depends on specialized molecules including ankyrin$_G$ and caspr. Ankyrin$_G$ interacts with $Na^+$ channels, $Na^+K^+$-ATPase, L1 and NCAM sequestering them to the node. Caspr localizes to the axoglial junctions responsible for the specializations at the node. The insulin receptor (IR) is localized to paranodal membranes, suggesting that insulin signaling is critical to nodal activity. In addition, insulin signaling is potentiated by C-peptide. *In vitro* studies suggest an interaction between caspr and Pi3K via a SH3 domain. We hypothesize that these molecules are altered in type I neuropathy and that insulin and/or C-peptide deficiencies underlie these changes. Immunoblotting (sciatic nerve) and semi-quantitative RT-PCR (DRG) reveal no differences in ankyrin$_G$ and its associated molecules between control; C-peptide treated (75 ng/kg/d), and untreated diabetic BB/W-rats at 2 and 8 mo. Ankyrin$_G$ is post-translationally modified by O-linked N-acetylglucosamine (O-GlcNAc). Immunoprecipitation of ankyrin$_G$ in SH-SY5Y cells treated with C-peptide (3.3 nM), insulin (4.0 nM), or both for 2 hr showed increased O-GlcNAc modification of ankyrin$_G$ with insulin only. Cells exposed for 20 hr showed decreased O-GlcNAc modification by insulin alone but was enhanced with C-peptide. Therefore, C-peptide potentiates the effect of insulin long term (20 hr) but blunts its effects short term (2 hr). Immunoblotting (sciatic nerve) and semi-quantitative RT-PCR (DRG) revealed no difference in the expression level of caspr at 2 mo. At 8 mo. caspr protein expression was decreased in diabetics but increased in C-peptide treated animals. Immunohistochemical studies show that caspr is laterally displaced from the paranode in diabetic rats at 2 mo, which is prevented by C-peptide. These findings suggest that localization and post-translational modifications may be more important than alterations in the expression of these nodal molecules in type I diabetic neuropathy.

### Reduced Protein Kinase C Activity in the Peripheral Nerve of Diabetic Mice Overexpressing Human Aldose Reductase

SHIN-ICHIRO YAMAGISHI, SATOSHI SUGAI, NOZOMI MASUTA, KAORI OKAMOTO and SOROKU YAGIHASHI

*Department of Pathology, Hirosaki University School of Medicine, Japan*

To clarify the relationships between activation of polyol pathway and altered protein kinase C(PKC) activity in the peripheral nerve, we examined PKC activity in the peripheral nerve of diabetic transgenic mice (Tg) that overexpress human aldose reductase. We evaluated the expressions of PKC isozymes by Western blot analysis. Littermate mice (LM) were used as a control. The mice at 8 weeks of age were made diabetic by i.p. injection of streptozotocin and followed for 12 weeks.

At end, body weight and levels of hyperglycemia were comparable between diabetic Tg and LM. There was 1.5 fold increase in nerve sorbitol levels in diabetic Tg compared with diabetic LM. Nerve PKC activity in membrane fraction in diabetic Tg was reduced by 28% ($p < 0.05$ vs. LM, Tg and diabetic LM). There was no significant change of PKC activity in the cytosolic fraction among groups. Western blot analysis revealed that the expression of membrane PKC $\alpha$ was decreased by 32% in diabetic Tg. While that of cytosolic PKC $\alpha$ increased 1.8 fold in the diabetic Tg.

These findings indicate that PKC activity is reduced in the diabetic nerve in association with increased flux of polyol pathway. Translocation of PKC $\alpha$ from the membrane to cytosol may account for the reduced PKC activity in the nerve.

### Proliferative Retinal Changes in Diabetic Rats (WBN/Kob)

T. MATSUURA, K. OZAKI and I. NARAMA

*Research Institute of Drug Safety, Setsunan University*

Proliferative retinopathy, one of the most common causes of blindness in severely diabetic human patients, were detected in aged male rats of WBN/Kob strain with persistent diabetic conditions by a perfusion technique using liquid silicone rubber and histological examination. In four of six diabetic rats, neovascularization occurred in the cloudy vitreous body or on a part of the retina on both sides at 24 months of age. These newly developed vessels extended and originated from the vessel near the optic disc. Although hyperglycemia and glucosuria lasted from 14 months to 23 months of age, they began to recede from about 17 months of age, and glucose returned to the normal level at 24 months of age. WBN/Kob rats are considered to be the first animal model that shows a successful induction of diabetic proliferative retinal changes. These animals may provide a suitable model for a classification of the pathogenesis and an evaluation of the relationship between diabetic retinopathy and blood sugar regulation.

### Interrelation between TRP Metabolism, MET Metabolism and $ZN^{2+}$ in Diabetic Rats

Y. SHIBATA, S. TANAKA, O. SHIBATA, M. NAKATSUKA and M. ISHIZU

*AICHI Medical University, Nagakute, Aichi, Japan*

In diabetic rats ($V.B_6$ deficient rats, STZ-diabetic rats and alloxan rats), liver kynureninase activity decreased. After taurine administration, this condition recovered.

On the other hand, after administration of $Zn^{2+}$, the content of zinc in brain, increased compared from that of liver and kidney.

And also zinc content especially increased in strain stem of $V.B_6$ deficient rats.

Cystathionine syntase and lyase may have the similar function as kynureninase. $Zn^{2+}$ is very effective to neurotransmitter.

**The ratio of Zinc Content in Brain**
**(Y. Sotokawa, O. Shibata, and S. Kobayashi)**

| | Control | Zinc Adm. Group |
|---|---|---|
| Brain | 100.0 ± 13.8 | 369.0 ± 93.1 |

**Zinc Content in $V.B_6$ Deficient Rats**
**(O. Shibata, M. Kimura, Y. Itokawa, M. Ishikawa, and H. Kikuchi)**

| | Control | $V.B_6$ Def |
|---|---|---|
| Brain Stem | 14.9 ± 1.6 | 16.8 ± 0.4 |

### Histochemical and Morphometrical Analysis of Skeletal Muscle in Spontaneous Diabetic WBN/Kob Rat

KIYOKAZU OZAKI, TETSURO MATSUURA and ISAO NARAMA

*Research Institute of Drug Safety, Setsunan University, Osaka, Japan*

Spontaneous diabetic WBN/Kob rats develop diabetic peripheral neuropathy characterized by primary segmental demyelination and secondary axonal degeneration. The objective of this study is to evaluate the histochemical and morphometric characteristics of the lesions of skeletal muscles innervated by the affected nerves in diabetic rats. Ten rats each comprised the following groups: 24-month-old males diabetic for less than 12 months, 10-month-old prediabetic males, 24-month-old nondiabetic females, and 10-month-old nondiabetic females. The soleus (SOL), extensor digitorum longus (EDL) and biceps femoris (BF) muscles were studied by light and electron microscopy, including histochemical and morphometric analyses. Muscle weight was reduced with age to a remarkable degree in diabetic BF and EDL. Dispersed atrophy of muscle fiber was observed in type 2a fibers of BF and EDL, and type 2c fibers of SOL, and the incidence was higher in diabetic rats. Multi-core, myofibrillar disorientation and an increased number of central nucleus of SOL, along with connective tissue proliferation of BF perimysium were noted in diabetic rats. The fiber population and type of composition varied with age, but no remarkable changes attributable to diabetic conditions were observed. Electron microscopically, an abnormal arrangement of myofibrils, a number of myelin figures, mitochondrial swelling and lysis of mitochondrial cristae were seen in diabetic rats. However, the neuromuscular junction and capillaries were intact. These findings indicate that the diabetic skeletal muscle lesion in WBN/Kob rats was mainly myogenic in nature, and was aggravated by the age-related change.

### Stirol Foam Balls Placed in the Stomach Prevent the Obesity and Improve the Carbohydrate Metabolism in OLETF Rats

EIJI OHMURA, HIROMI BANBA, MASAKO YAZAWA, ATSUKO TSUCHIDA, DAIYA HOSAKA, SINNYA MINAGAWA, CHIKA IHARA, YASUO IMAI and SHOJI KAWAZU

*Saitama Medical Center, Saitama, Japan*

The effect to intra-gastric installation of the stirol foam balls on the changes of body weight and carbohydrate metabolism was investigated using OLETF rats. Male OLETF rats (350–400 g, Ohtsuka Pharm. Inst.) were anesthetized with ether and three balls (1.2 cm in diameter) were placed in the stomach (SF group; $n = 9$). No ball was placed in the sham operated rats (C group; $n = 10$). Body weights of SF group were decreased by 12% (399 ± 11 g vs. 453 ± 8 g) and 14% (501 ± 11 g vs. 580 ± 13 g) after 4 and 12 weeks of operation, respectively. Intra-peritoneal glucose tolerance test revealed that the blood glucose levels were significantly suppressed in SF group after 30 and 60 min., although fasting blood glucose levels were not different. In OLETF rats (610–690 g) developed diabetes (FBS > 140 mg/dl), the installation of the balls was effective to reduce blood glucose level. In conclusion, intragastric installation of the stirol foam balls prevents the increment of body weight and improves the carbohydrate metabolism.

### Comparison of Glucokinase Activities in the Peripheral Leukocytes between Dogs and Cats

AKIO TANAKA, TSUKIMI WASHIZU, TOSHINORI SAKO and TOSHIRO ARAI

*Department of Veterinary Science, Nippon Veterinary and Animal Science University, Musashino, Tokyo, Japan*

Activities of hexokinase (HK), glucokinase (GK) and pyruvate kinase (PK) were measured in peripheral leukocytes (WBC) of dogs and cats. Dog WBC showed GK activities and specific fragment containing region including glucose- and ATP-binding domains of GK as determined with RT-PCR. However, in cat WBC, the activities and specific fragment of GK were absent. After fasting, the activities and gene expression of GK decreased greatly in the dog WBC. The cat WBC had significantly higher activities of HK and PK than dog WBC. There is a clear difference in glycolytic enzyme activities in WBC between dogs and cats. Activities and mRNA quantity of GK are considered to be varied with changing of the metabolic conditions, *e.g.*, fasting or refeeding. And cDNA sequence of GK is analyzed in WBC from normal and diabetic dogs.

### Activities of Enzymes in the Malate-Aspartate Shuttle in the Peripheral Leukocytes in Diabetic Dogs and Cats

TSUKIMI WASHIZU, MIHOKO TAKAHASHI, AKIO TANAKA and TOSHIRO ARAI

*Department of Veterinary Science, Nippon Veterinary and Animal Science University, Musashino, Tokyo, Japan*

The activities of the enzymes involved in the malate-aspartate shuttle and the expression of malate dehydrogenase (MDH), a late limiting enzyme in the NADH shuttle, were determined to investigate the differences in this shuttle system in peripheral leukocytes in dogs and cats. There were no significant differences between dogs and cats in plasma glucose, IRI and FFA concentrations. The activities of MDH and glutamate dehydrogenase in canine leukocytes were significantly higher than in feline leukocytes. High activities of MDH in canine leukocytes were confirmed by RT-PCR analysis on the total RNA extracted from leukocytes. It was concluded that there were significant differences between dogs and cats in the NADH shuttle system. Moreover, in the leukocytes of diabetic dogs, MDH activities and gene expression decreased remarkably.

### Effect of Diabetes on Prednisolone Disposition in the Rats

RYO MURATA[a], YUKO ICHIHARA[a], AYAKO SUDO[a], TADAHIRO YOSHIDA[a], YORIHISA TANAKA[a], SUSUMU SUZUKI[b] and TAKAYOSHI TOYOTA[c]

*[a]Department of Pharmaceutics, Tohoku Pharmaceutical University, Sendai, Japan, [b]Department of Metabolism and Diabetes, Tohoku University Graduate School of Medicine, Sendai, Japan, [c]Tohoku Rousai Hospital, Sendai, Japan*

We examined pharmacokinetic study of prednisolone in two types of diabetic rats, streptozotocin-induced diabetes (STZ) and GK rats. Blood samples were collected from the jugular vein after i.v. or p.o. administrations of prednisolone and determined the concentrations of prednisolone and prednisone (active metabolite) by HPLC. There were no significant differences in the pharmacokinetics of prednisolone and prednisone after i.v. administration in the two different types of diabetic rats compared with those of the control. After p.o. administration of prednisolone, however, the pharmacokinetic parameters of $T_{max}$ and $C_{max}$ of prednisolone and prednisone after p.o. administration of prednisolone in GK rats were changed compared with those of STZ and the control rats. It is suggested that the absorption and metabolic processes of prednisolone might be influenced by the diabetic state in GK rats.

### Characteristics of WBN/Kob-*Lepr^fa*: A New Diabetic Congenic Rat Strain with Obesity

T. AKIMOTO[a], K. NAKAMA[a], M. SUGAWARA[b] and K. KOMEDA[c]

*[a]Nippon Medical School, Laboratory Animals, [b]Nippon Veterinary and Animal Science University, Animal Biochemistry, [c]Tokyo Medical University, Animal Research Center*

WBN/Kob male rats spontaneously develop severe pancreatitis and diabetes without obesity at approximately 20 and 37 weeks of age, respectively. The leptin receptor fatty gene ($Lepr^{fa}$) mutation is a recessive mutation of the leptin receptor. All the homozygous ($Lepr^{fa}/Lepr^{fa}$) rats develop severe obesity, hyperinsulinemia, yet euglycemic, as shown in the Zucker fatty rat. In this study, we describe the production and histological characterization of a congenic rat strain carrying the leptin receptor ($Lepr^{fa}$) mutation on a WBN/Kob background by successive backcrosses (N6). Genetic polymorphism at the *Lepr* locus was detected by the PCR-RFLP assay. In generation N3F1, 49 male and 36 female rats were obtained from mating heterozygous ($+/Lepr^{fa}$) male and female. All the $Lepr^{fa}/Lepr^{fa}$ rats (11 male and 9 female) from this mating were obese. Moreover, all obese male rats developed diabetes at 11–13

weeks of age, but only three out of the nine obese female rats developed diabetes by 13 weeks of age. We did not observe any signs of obesity and diabetes in +/*Lepr*$^{fa}$ and +/+ rats at 13 weeks old in both sexes. All male rats of backcross generation N4 showed signs of severe pancreatitis at 20 weeks old. We conclude that the congenic rat strain, WBN/Kob-*Lepr*$^{fa}$, would be an efficient tool for research on the pathogenesis of type 2 diabetes with obesity.

### Morphological Observations on the Pancreatic Islets of Spontaneous Diabetic KKAy Mice

YASUAKI ICHIKAWA and KAZUO YAMASHITA

*Department of Anatomy, Nippon Medical School, Tokyo, Japan*

The pancreatic islets of diabetic KKAy mice (8 and 12 weeks, male) and nondiabetic age- and sex-matched C57BL/6 mice were morphologically examined. This diabetic animal model is characterized by early onset and prolongation of severe hyperinsulinemia and hyperglycemia. KKAy 8 week's mice showed hypertrophied islets occupied mostly by insulin secreting B cells. B cells in the cores of the islets of the KKAy 12 week's mice were degenerated whereas those in their peripheries were hyperplastic. The degenerated B cells showed swollen mitochondria and glycogen granule deposits. An increase collagen fibers also were found around stagnate capillaries in the cords of the islets. Interestingly many insulin positive cells were detected in the epithelium of the common bile and pancreatic ducts, suggesting differentiation of new B cells from their possible ductual stem cells.

### Quantitative Trait Locus Analysis for Chronic Pancreatitis in (BNXWBN)F2 Progeny

A. TSUJI[a], T. NISHIKAWA[a], M. MORI[b], I. NISHIMORI[c], K. SUDA[d] and M. NISHIMURA[e]

[a]*Inst. Exp. Animals, Hamamatsu University School of Medicine,* [b]*Department Aging Angiology, RC. Aging & Adaptation, Shinshu University School of Medicine,* [c]*First Intern. Med., Kochi Medical School,* [d]*Pathol., Juntendo University School of Medicine,* [e]*Inst. Exp. Animals, Nagoya University School of Medicine*

A causative gene mutation is still undefined in around half of patients with hereditary pancreatitis and no genetic factor has been identified in most patients with sporadic chronic pancreatitis. To identify pancreatitis-associated genes, we performed a quantitative trait locus (QTL) analysis for the traits of chronic pancreatitis in WBN/Kob rats, known as model animal for chronic pancreatitis. We identified two significant ($P < 0.001$) QTLs on chromosomes 7 (LRS = 17.1), and X (LRS = 17.4). These QTLs were located on completely different chromosomal regions from those of causative genes that have been reported for human chronic pancreatitis; *PRSS1*, *CFTR* and *SPINK1*. For these QTLs, prevalences of the WBN/Kob allele significantly increased in the rats with chronic pancreatitis. These findings indicate that chronic pancreatitis in WBN/Kob rats is controlled by multiple genes and a genetic analysis in WBN/Kob rats might be useful for gene targeting for human chronic pancreatitis.

### Small Fiber Neuropathy in Impaired Glucose Tolerant (IGT) GK Rats

A. A. F. SIMA[a], Y. MURAKAWA[a], W. ZHANG[a], T. BRISMAR[b], C. G. OSTENSON[b] and EFENDIC[b]

[a]*Wayne State University, Detroit, MI 48201, USA,* [b]*Karolinska Institute, Stockholm, Sweden*

The functional and structural neuropathy was examined in 18 mo IGT GK rats. IGT rats showed decreased body weight (454 ± 31 vs. 627 ± 80 g; $p < 0.001$), increased blood glucose levels (4.4 ± 1.3 vs. 3.2 ± 0.4 mmol/l; $p < 0.02$) and impaired glucose tolerance ($p < 0.001$). IGT rats showed decreased nerve conduction velocity (49.8 ± 51 vs.

61.8 ± 4.9 m/sec; $p < 0.001$). Sural nerve morphometry showed increased fascicular area ($p < 0.01$), normal fiber number (N.S) and decreased fiber density ($p < 0.001$), indicating endoneurial edema. Myelinated fiber parameters showed decreased axon/myelin ratio ($p < 0.05$), normal mean axonal area (N.S), increased mean fiber area ($p < 0.001$) and myeline area ($p < 0.01$). These data suggest that small myelinated fibers are preferentially affected with preservation of large myelinated fibers. This was confirmed by fiber size distribution histograms which showed a significant relative increase in fibers >45 $\mu m^2$ in diabetic rats (42.6 ± 2.9 vs. 29.8 ± 3.8%; $p < 0.001$). These findings differ from those in type 1 or type 2 diabetic neuropathy (STZ-rat; BB/W-rat), which is characterized by fiber loss, axonal atrophy affecting all fiber categories and lack of endoneurial edema in the chronic stage of neuropathy. These findings are consistent with those in IGT humans. In conclusion IGT GK rats show a mild neuropathy after 18 mo of IGT, which affects preferentially small myelinated fibers.

### Hyperglycemia Induced Intracellular Protein Glycation and Activation of ERK1/2 in Ischemic and Reperfused Rat Heart

GUANG XU, EN TAKASHI, ZENYA NAITO, TOSHIYUKI ISHIWATA, MUNEHIRO YOKOYAMA, NOKUTAKA YAMADA and GORO ASANO

*Department of Pathology, Nippon Medical School, Tokyo, Japan*

Hyperglycemia can occur intracellular protein glycation, and resulting altered their structure and function. Extracellular signal-regulated kinase (ERK) 1/2 is important intracellular kinase in the signal transduction and plays a key role for the survival in ischemic myocardium. This present study was designed to clarify the ERK 1/2 activation whether or not correlated with intracellular AGEs accumulation in heart. Rats were administrated with streptozotocin (STZ, 60 mg/kg, ip) induced diabetes mellitus after 4 and 20 weeks (W). These diabetic or no diabetic rats were subjected to 30 min of regional ischemia and 120 min of reperfusion (I/R). Western blot analysis showed the activation of ERK 1/2 was increased in 4 W after STZ treatment heart, and the condition of I/R was more increased ERK 1/2 activation. On the other hand, the activation of ERK 1/2 was decreased with 20 W after STZ injection, and ERK 1/2 reaction was not sensitive to I/R. Immunohistochemically, advanced glycation end products (AGEs) were accumulated in the cardiomyoctyes of diabetic rat. These results demonstrate that diabetes mellitus reduces the ischemic sensitivity with induced ERK activation due to intercellular protein glycation.

### Effect of Zinc Acetate on Gold Thioglucose Produced Obesity

REIKO NATSUKI

*Department of Toxicology, Faculty of Pharmaceutical Sciences, Setsunan University, Hirakata, Osaka 573-0101, Japan*

The effect of zinc acetate treatment in gold thioglucose produces non insulin-dependent diabetes mellitus (GTG) via obesity in mice was investigated. Mice were injected i.p. with either saline (control) or GTG (500 mg/kg) that produces obesity, and induces diabetes mellitus. Both control and GTG mice were given 0.3% zinc acetate in rodent chow powder for 5 months after GTG injection. In the present study, glucose and glycoprotein (HbA1c) levels in the blood, insulin, cholesterol (Ch) and triglyceride (TG) levels in the plasma were determined. Body weight was measured at regular intervals after GTG injection and taken zinc acetate food until the mice killed on the 5 months. In control mice, zinc acetate did not have a significant effect on body weight, blood-glucose amd-HbA1C, and insulin, Ch and TG in plasma. In contrast, in GTG treated mice, zinc acetate decreased markedly those which were elevated significantly in GTG treated mice than that of control. *In vitro*, using intestinal microsomes, zinc acetate inhibited α-glucosidase activity with several substrates such as sucrose and carbohydrates. Zinc acetate does exert an inhibitory effect of α-glucosidase activity in gastrointestinal tract, and also, rerate to glucose-responsive neurons. It is concluded that zinc acetate may be able to improve the deterioration in the glycemic controls and inhibit the development of diabetic in GTG mice.

### Accumulation of Body Fat and Intra-Muscular Fat in Pioglitazone-Treated OLETF Rats and Its Relation to the TNF-α mRNA Expression

YUTAKA MORI, HIDEAKI KOMIYA, NAOYUKI KUROKAWA, YOSHIKO INABA, JUNICHI YOKOYAMA and NAOKO TAJIMA

*Department of Int. Medicine, National Higashi-Utsunomiya Hospital, Tochigi, Japan*

We investigated the influence of pioglitazone (P) on the TNF-α mRNA expression in the white adipose tissue (WAT). Male OLETF rats aged 8 weeks were randomly divided into 4 groups including P group, glibenclamide (G) group, G+P group and control (C) group for 12 weeks of observation. The significant increases in the body weight, the WAT weights and the triglyceride content in skeletal muscle were observed in P and G+P groups. The TNF-α mRNA levels in the WAT of P and G+P group decreased significantly compared with those in control and G groups, respectively. Morphometric analysis of adipocyte distribution along with their size indicated that G increased the population of larger-sized adipocytes, and P increased the population of smaller-sized adipocytes and decreased the population of larger-sized adipocytes in WAT. It was possible that pioglitazone could decrease the TNF-α mRNA expression in WAT and reduce insulin resistance by reducing the size of adipocytes. On the other hand, pioglitazone was also considered to affect the adipocytes in skeletal muscle.

### The Improvement of Insulin Secretion after Sugar Load with Nateglinide in OLETF Rats: Difference in Response with Aging

YUTAKA MORI, HIDEAKI KOMIYA, YOSIKO INABA, JUNICHI YOKOYMA and NAOKO TAJIMA

*Department of Int. Medicine, National Higashi-Utsunomiaya Hospital, Tochigi, Japan*

Nateglinide is a new fast-onset, short-acting hypoglycemic agent that directly stimulates the pancreatic β cells. We investigated the effect of nateglinide on insulin secretion after sugar load in OLETF rats whose insulin secretion in response to glycemic stimulus became delayed and decreased with advancing age. Male OLETF rats aged 8, 16 and 24 weeks were administered nateglinide (50 mg/kg) or methylcellulose as the control just before sucrose load of 2.5 g/kg. Blood samples were obtained from retroorbital sinus before sucrose load, 30 min after, 60 min after and every 60 min thereafter until 360 min. At the age of 8 and 16 weeks, plasma glucose levels at 30, 60 min after sucrose load were significantly decreased on nateglinide. On nateglinide, the plasma IRI levels were significantly increased at 30 min after sucrose load in 8-week-old rats and at 60 min after sucrose load in 16-week-old rats. At the age of 24 weeks, plasma glucose levels were significantly decreased at 30, 60 and 120 min after sucrose load on nateglinide, while no significant increase in plasma IRI levels was observed on nateglinide. Our results indicate that the effect of nateglinide on insulin secretion in response to glycemic stimulus is likely to depend on the individual insulin secretion capacity.

### Effect of Glimepiride on the TNF-α mRNA Expression in White Adipose Tissue and Its Relation to the Change in Cellularity: Comparison with Glibenclamide

YUTAKA MORI, HIDEAKI KOMIYA, NAOYUKI KUROKAWA, YOSHIKO INABA, JUNICHI YOKOYAMA and NAOKO TAJIMA

*Department of Int. Medicine, National Higashi-Utsunomiya Hospital, Tochigi, Japan*

We investigated the influence of glimepiride on the TNF-α mRNA expression in the white adipose tissue (WAT) in comparison to the influence of glibenclamide. Male OLETF rats aged 8 weeks were randomly divided into 3

groups including glimepiride group, glibenclamide group and control group for 12 weeks of observation. The plasma IRI and triglyceride levels were significantly increased in glibenclamide group compared with those in the controls. The TNF-α mRNA levels in the WAT of glibenclamide group were significantly increased compared with those in the controls, while no significant increase was observed in glimepiride group. Morphometric analysis of adipocyte distribution along with their size indicated that glibenclamide increased the population of larger-sized adipocytes, while glimepiride did not. Our results indicate that glibenclamide is likely to increase the TNF-α mRNA expression in WAT and accelerated insulin resistance by increasing the size of adipocytes in OLETF rats. On the other hand, glimepiride was considered not to increase the TNF-α mRNA expression in WAT and not to accelerate insulin resistance because of its mild insulin stimulating action.

### Ethyl Acetate Extract from Licorice Root Displays Anti-Diabetic and Anti-Obesity Effect on KK-Ay Mice

KAZUMA TAKAHASHI[a], KAKU NAKAGAWA[b], TATSUMASA MAE[b], TERUO KAWADA[c] and JO SATOH[a]

[a]*Division of Molecular Metabolism and Diabetes, Tohoku University, Sendai, Japan,* [b]*Kaneka Co., Osaka, Japan,* [c]*Division of Applied Life Sciences, Kyoto University, Japan*

Licorice root (*Glycyrrhiza*) has been used as food, and for medicinal purposes as analgesics, antitussives, and expectorants for centuries. Glycyrrhizin, the major aqueous component, now serves as an anti-inflammatory medicine. The oleaginous components are usually used as an antioxidant additive, however, other various pharmacological effects are possibly expected, because of unique chemical structure of flavonoids contained.

In this study, we test ethyl acetate extract from licorice for insulin sensitizing effect on KK-Ay mice, an animal model for diabetes mellitus with insulin resistance.

Thirteen-week-old make KK-Ay mice were administered once daily with the extract (250 mg/kg/day) for 14 days. The administration significantly reduced non-fasting blood glucose concentration (410.2 ± 100.1 mg/dl, $n = 6$, $p < 0.03$) and epididymal fat weight (1.51 ± 0.109 g, $n = 6$, $p < 0.01$) compared to the controls (490.3 ± 32.85 mg/dl and 1.85 ± 0.198 g, respectively), without changing food intake and body weight. The fasting plasma insulin concentration was comparatively lower in the administered group, but not significantly.

The ethyl acetate extract from licorice ameliorated hyperglycemia in KK-Ay mice possibly via a mechanism involving reduction of the visceral adipose tissue.

### Increased Expression of the Uncoupling Protein-2 and 3 Gene in Obese, Hyperglycemic Mice Induced by Goldthioglucose

K. IMAMAKI[a], S. TAKEI[a], N. TANE[a], C. ISHII[b], S. KATAYAMA[a] and K. NEGISHI[c]

[a]*Department of Medicine, Saitama Medical School,* [b]*Department of Medicine, Saitama National Hospital,* [c]*Neigishi Clinic*

Uncoupling protein (UCP)3 and UCP2, mitochondrial carrier proteins with high homology to UCP1 which have been implicated in the regulation of energy metabolism. Particularly UCP3 gene is expressed abundantly in the skeletal muscle, while UCP2 gene is detected in the white adipose tissue with diffuse localization throughout the body. UCP3 and UCP2 could also be involved in the reguration of energy balance.

Since obesity is associated with disturbed energy and glucose metabolism, we tested the hypothesis that UCPs gene expressions are involved in obese, hyperglycemic mice which induced by goldthioglucose (GTG).

Obese, hyperglycemic mice were induced by injection of GTG at the age of 6 weeks. Body weight, plasma glucose and insulin levels were significantly increased at the age of 11 weeks in GTG mice comparing with lean control mice. Pancreatic beta cell mass was also increased in GTG mice at the age of 16 weeks.

UCP2 mRNA levels were significantly increased by 5.2-fold (16 weeks) and 2.9-fold (26 weeks) in the skeletal muscle from GTG mice as compared with age matched lean controls. UCP3 mRNA levels were also significantly higher by 2.7-fold (16 weeks) and 2.3-fold (26 weeks) in the skeletal muscle from GTG mice that that from age matched lean controls.

This present study supports the concept that UCP2 and UCP3 may play a role in the pathophysiology of obesity and glucose metabolism in GTG induced obese mice.

